# Supplementary figures and images for: Natural sounds can be reconstructed from human neuroimaging data using deep neural network representation
Source: PLoS Biol. 2025 Jul 23;23(7):e3003293. doi: 10.1371/journal.pbio.3003293 (PMC12313072; doi:10.1371/journal.pbio.3003293)

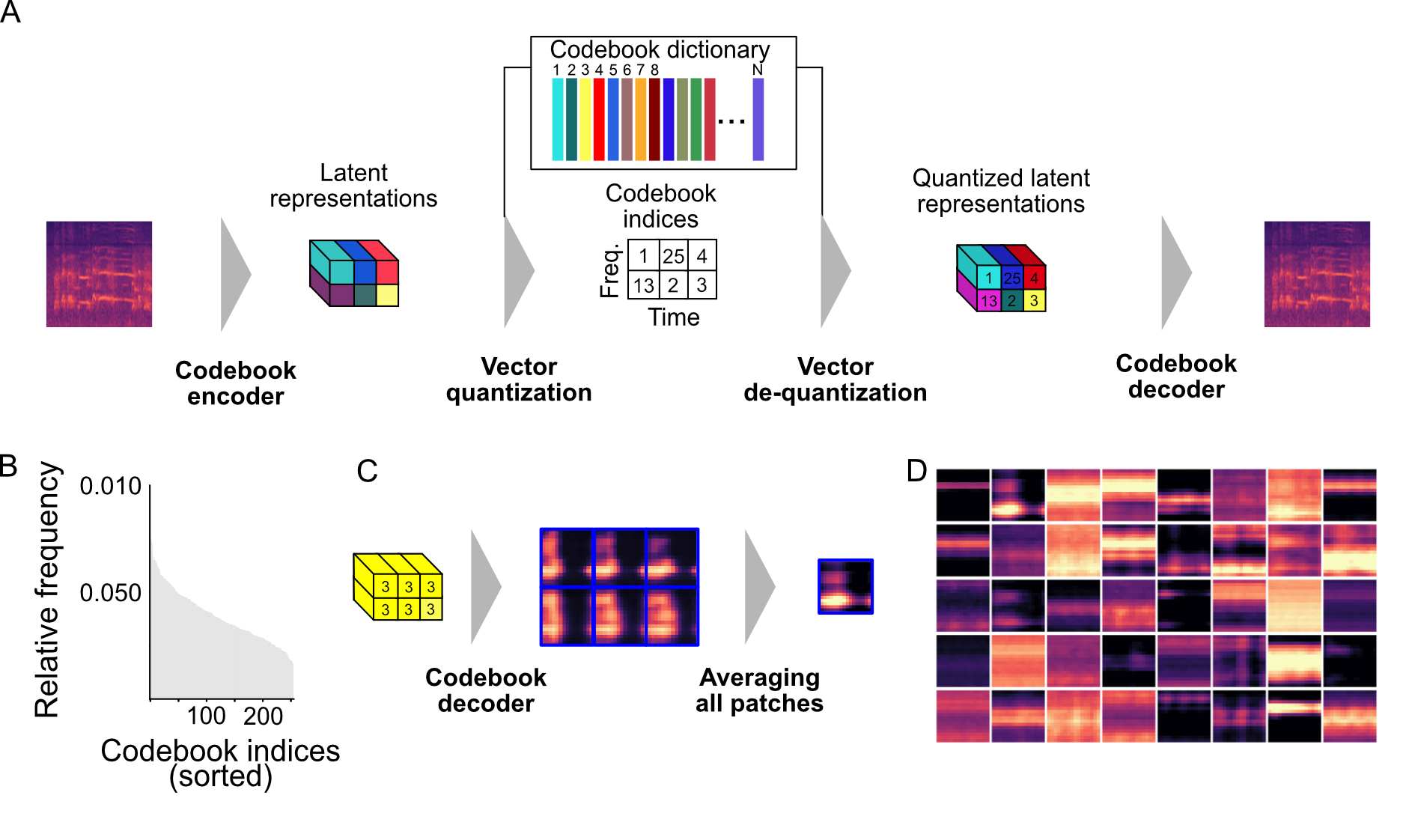

Supplement: S1 Fig — (A) Training. The SpecVQGAN (Spectrogram Vector Quantized Generative Adversarial Network) model, inspired by the Vector Quantized Variational Autoencoder (VQVAE) framework, is trained to encode spectrograms into compact representations and reconstruct them efficiently. The process begins with an input spectrogram that is transformed into latent representations by the codebook encoder. These representations are quantized into discrete codebook indices using a codebook dictionary, reducing the spectrogram’s dimensionality by a factor of 16. For example, a 4-s spectrogram with dimensions of (80 × 336) can be efficiently reduced to a (5 × 21) grid of codebook indices, though it is depicted as a (2 × 3) grid for simplified visual representation. The codebook indices are decoded back into quantized latent representations using the same dictionary and reconstructed into spectrograms by the codebook decoder. The model is trained using the VGGSound dataset, optimizing perceptual, adversarial, codebook, and reconstruction losses. (B) Histogram of codebook indices. A histogram displays the frequency distribution of codebook indices for the experimental stimulus dataset, generated using the SpecVQGAN model trained on the VGGSound dataset. The histogram showed a distributed use of codebook indices and maintained relative balance. (C) Interpretation of codes as spectrogram patches. To visualize the spectral and temporal patterns captured by individual codes, a set of indices consisting of identical codes is input into the codebook decoder, and the averaged segments highlight the characteristic patterns represented by each code. (D) Examples of SpecVQGAN codes. Among the 256 trained codes, 40 examples are illustrated, demonstrating the model’s capability to represent and reconstruct complex spectrograms. These findings highlight the ability of SpecVQGAN codes to capture intricate spectral and temporal features through various combinations of discrete codes. (TIFF) [file pbio.3003293.s001.tiff]

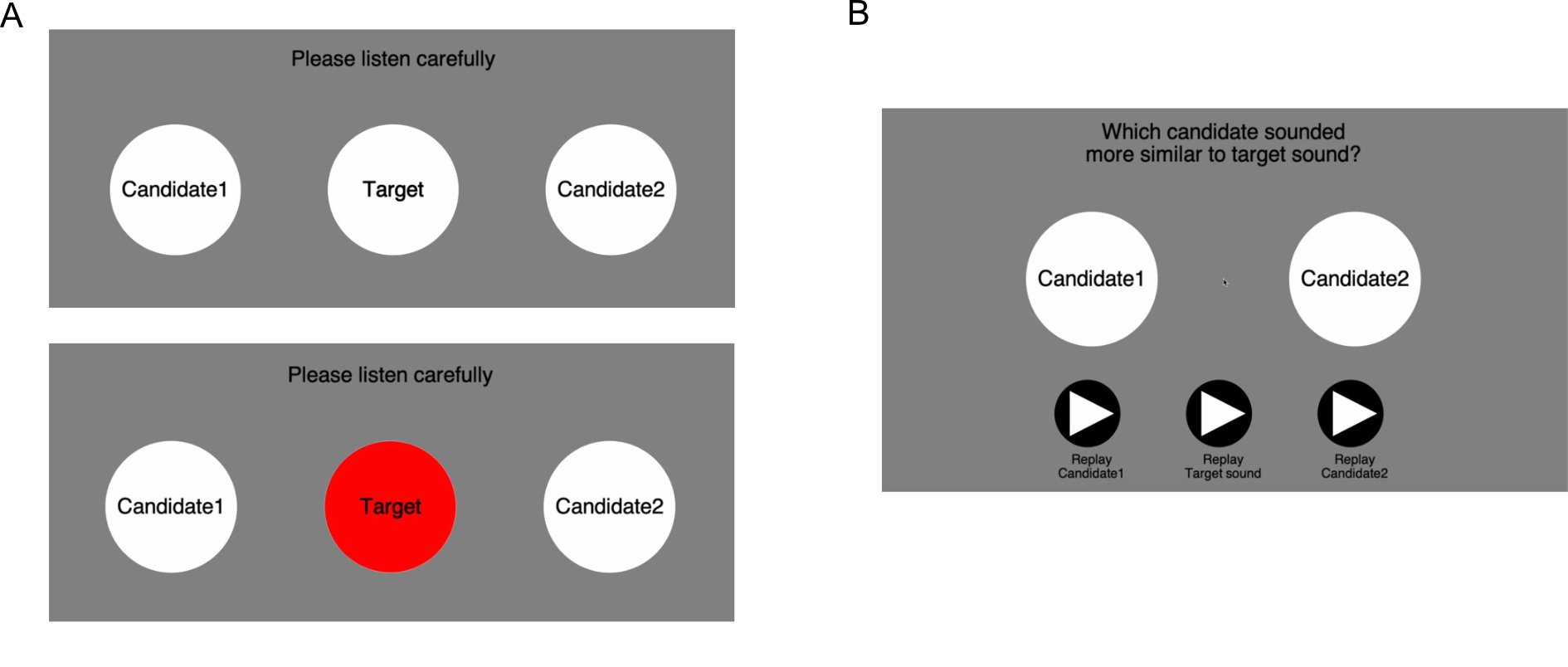

Supplement: S2 Fig — (A) Stimulus presentation phase. During the behavioral evaluation, participants were sequentially presented with three sounds: the reconstructed sound (target) and two candidate sounds—one corresponding to the true stimulus and the other a false candidate randomly selected from the test set. The currently playing sound was visually highlighted in red. Participants were instructed to assess which of the two candidate sounds was more similar to the target sound. (B) Selection phase. After listening to all three sounds, participants selected the sound they judged to be more similar to the target by clicking on the white circle corresponding to either Candidate 1 or Candidate 2. (TIFF) [file pbio.3003293.s002.tiff]

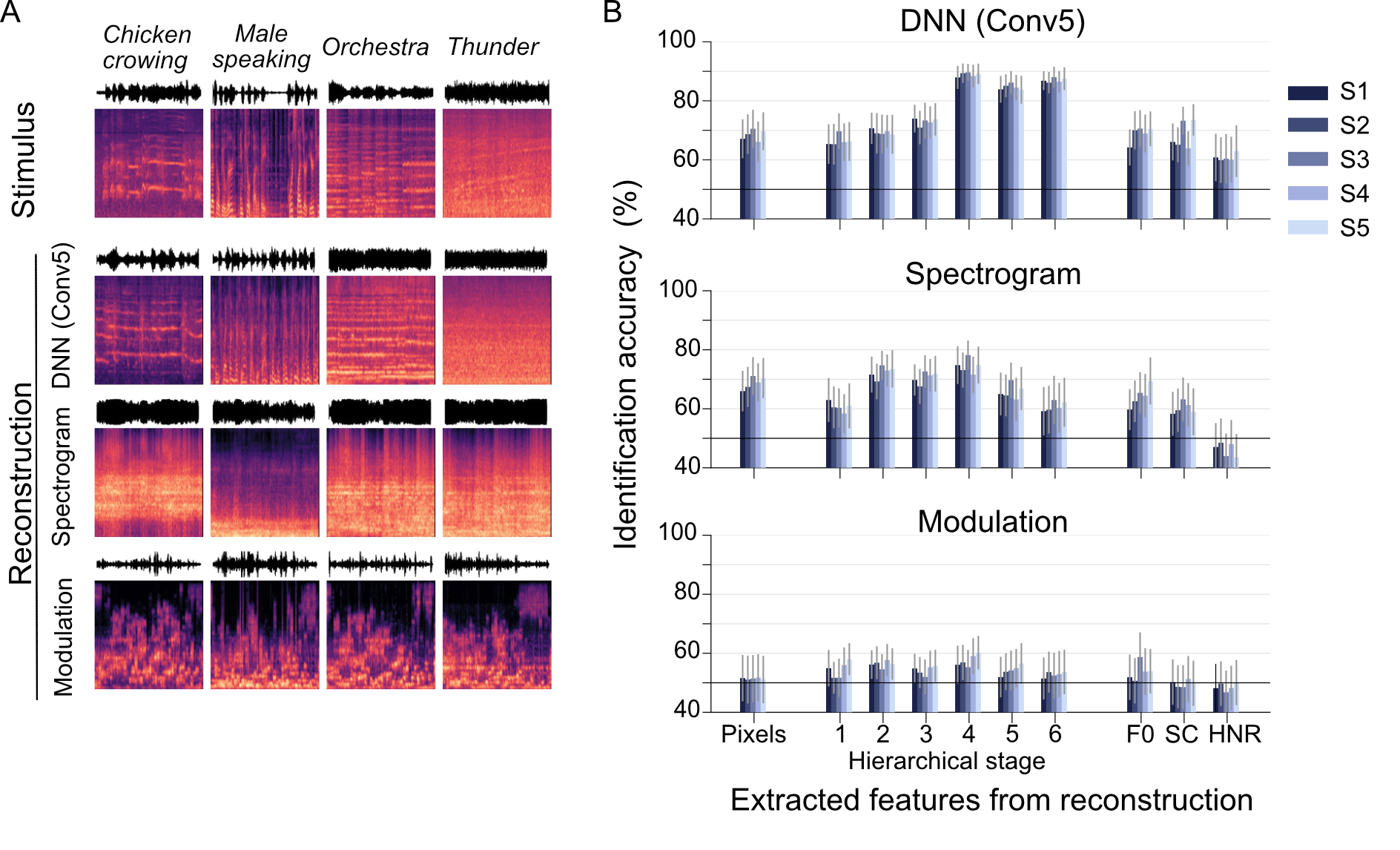

Supplement: S3 Fig — (A) Reconstructed spectrograms using various auditory features with conventional pipelines (Regions of interest (ROI): Auditory cortex (AC); for reconstructed sounds, see https://www.youtube.com/watch?v=KXcDOTOP0h8). The first row shows the original spectrogram of the presented sound. Rows two to four display reconstructions based on different auditory features decoded from AC. The spectrogram-based reconstruction method involves training a model to predict each pixel value of the spectrogram from fMRI responses, followed by conversion into sound waves using a spectrogram vocoder. The modulation feature-based reconstruction employs a two-step process: modulation features were transformed into a spectrogram and then converted into sound waves following a previous study [21]. Reconstructions derived from spectrogram features appear as temporally smooth versions of the original spectrograms. However, reconstructions from modulation features fail to capture distinct spectrotemporal patterns across different frequency ranges, resulting in diminished differentiation between stimuli. (B) Evaluation of reconstructed sounds. Each panel corresponds to a specific reconstruction pipeline. Each bar indicates mean identification accuracy for each subject, with error bars showing the 95% confidence interval (CI) based on 50 data points. Quantitative evaluations reveal that reconstructions using deep neural network (DNN) features outperform those based on other auditory features across most metrics. Spectrogram-based reconstructions achieve an identification accuracy of approximately 70% for pixel-level and lower hierarchical representations, with accuracy declining at higher hierarchical stages. Reconstructions based on modulation features identify stimuli at almost chance levels and showed inferior performance compared to DNN-based reconstructions. The data underlying this figure are provided in S2 Data. (TIFF) [file pbio.3003293.s003.tiff]

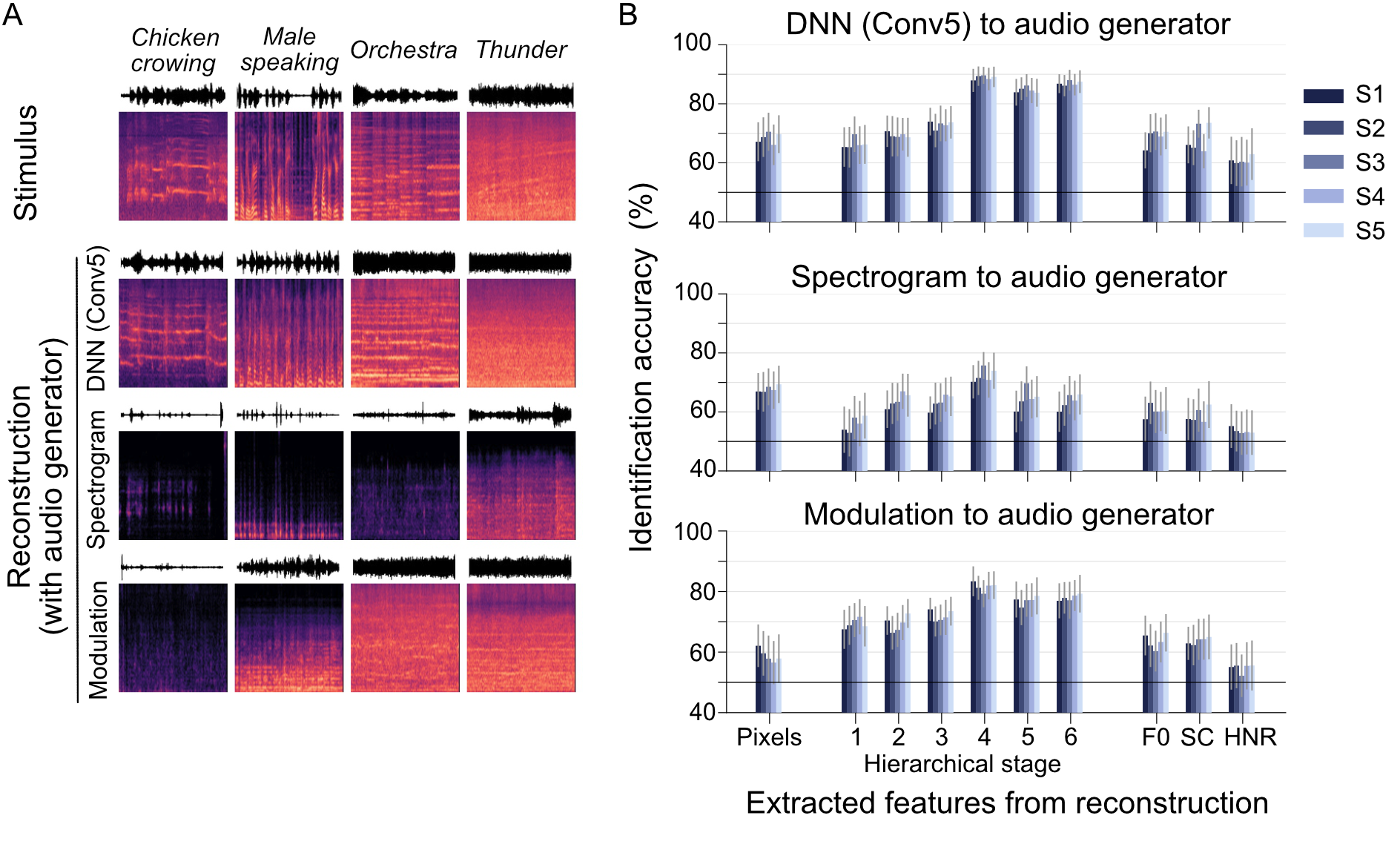

Supplement: S4 Fig — (A) Reconstructed spectrograms using various auditory features (ROI: AC; for reconstructed sounds, see https://www.youtube.com/watch?v=5ZH4upFgi0I). The first row presents the original spectrogram of the presented sound. Rows two to four display reconstructions generated from different auditory features decoded from AC. To examine the influence of different auditory features on reconstruction, only the input (DNN features) is replaced with spectrogram and modulation features while keeping other pipeline components unchanged. The audio generator is trained separately for each auditory feature. Notably, due to YouTube’s data availability, the modulation feature-based audio generator was trained on a dataset of 132,551 clips, whereas the DNN and spectrogram feature-based generators used 156,487 clips. Each decoded auditory feature is then used as input to its corresponding audio generator for reconstruction. Reconstructions derived from spectrogram and modulation features exhibit temporally smoothed patterns and partially captured spectrotemporal patterns across different frequency ranges. (B) Evaluation of reconstructed sounds. Each panel corresponds to a specific reconstruction pipeline. Bars indicate the mean identification accuracy for each subject, with error bars representing the 95% CI based on 50 data points. Spectrogram-based reconstructions achieve approximately 70% identification accuracy across most evaluation metrics. Reconstructions based on modulation features outperform those using spectrograms but showed lower performance compared to the proposed method using DNN features. Specifically, reconstructions based on modulation features exhibit inferior performance in higher hierarchical stages compared to DNN-based reconstructions. These results indicate that using DNN features for reconstruction outperforms other auditory feature-based approaches, even when utilizing the same reconstruction pipeline. The data underlying this figure are provided in S2 Data. [file pbio.3003293.s004.tiff]

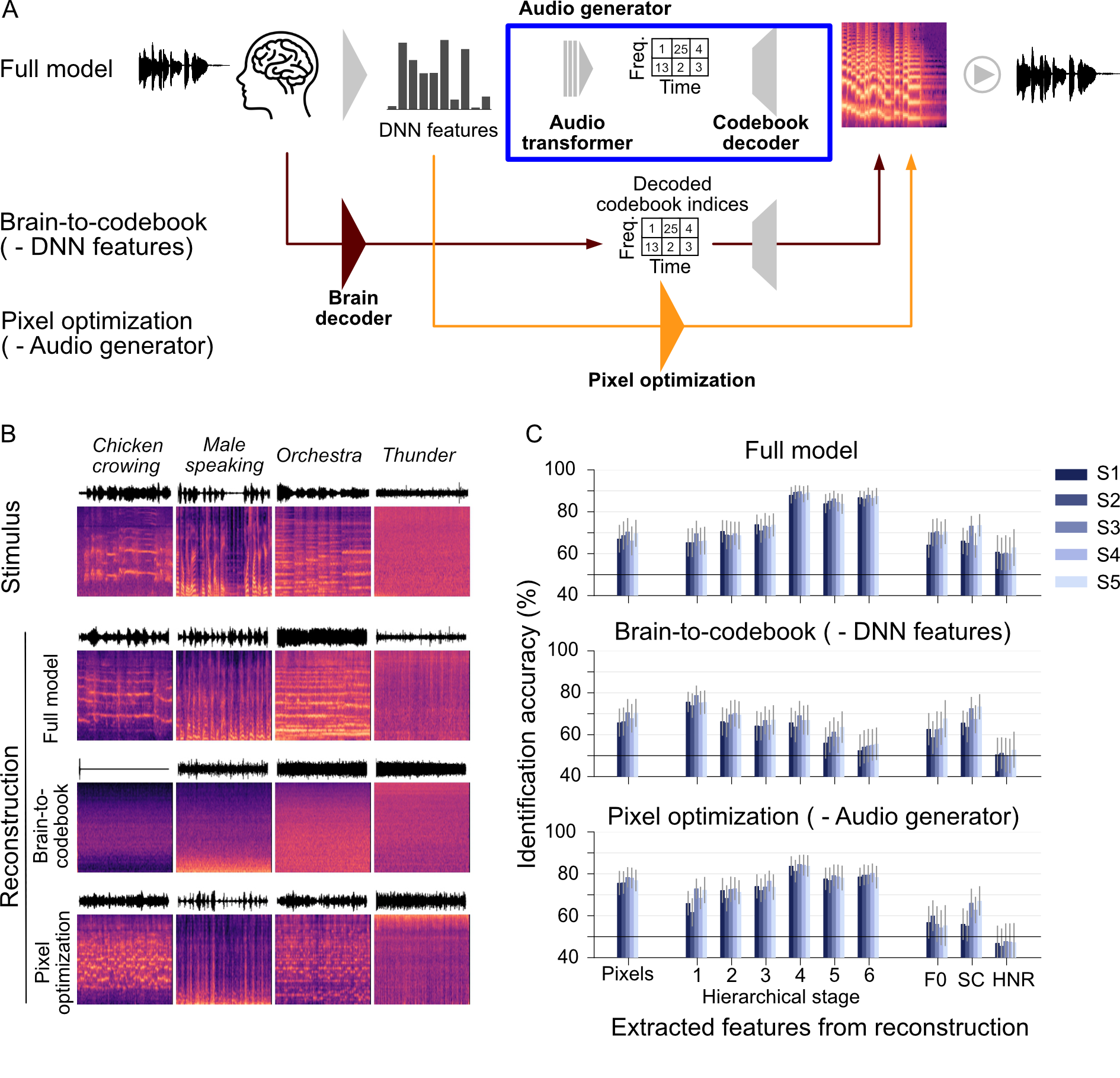

Supplement: S5 Fig — (A) Overview of reconstruction pipelines. The brain-to-codebook pipeline predicts codebook representations directly from fMRI responses (brown line), which are then transformed into spectrograms and sound waves via the codebook encoder and decoder. The pixel optimization pipeline iteratively adjusts spectrogram pixel values to match decoded DNN features inferred from fMRI responses (orange line). (B) Reconstructed spectrograms from different pipelines (ROI: AC; for reconstructed sounds, see https://www.youtube.com/watch?v=DFLsaLY4g64). The first row displays the original spectrograms of the stimuli, and subsequent rows show reconstructions from each pipeline. The brain-to-codebook pipeline produces temporally smoothed spectrograms, while the pixel optimization pipeline generates spectral patterns with specific frequency alignment but noticeable noise and limited temporal fidelity, resulting in texturally noisy outputs. (C) Evaluation of reconstructed sounds. Each panel represents mean identification accuracy per subject for a given reconstruction pipeline, with error bars denoting the 95% CI based on 50 data points. The brain-to-codebook pipeline achieves approximately 65% identification accuracy based on spectrogram pixels but showed declining performance at higher hierarchical stage. The pixel optimization pipeline performs better than the brain-to-codebook approach in both spectrogram pixel accuracy and higher hierarchical stage but still fell short of the proposed model. These results highlight that while both pipelines could reconstruct spectral patterns, only the proposed model consistently preserved perceptual qualities similar to the original stimuli. The data underlying this figure are provided in S2 Data. (TIFF) [file pbio.3003293.s005.tiff]

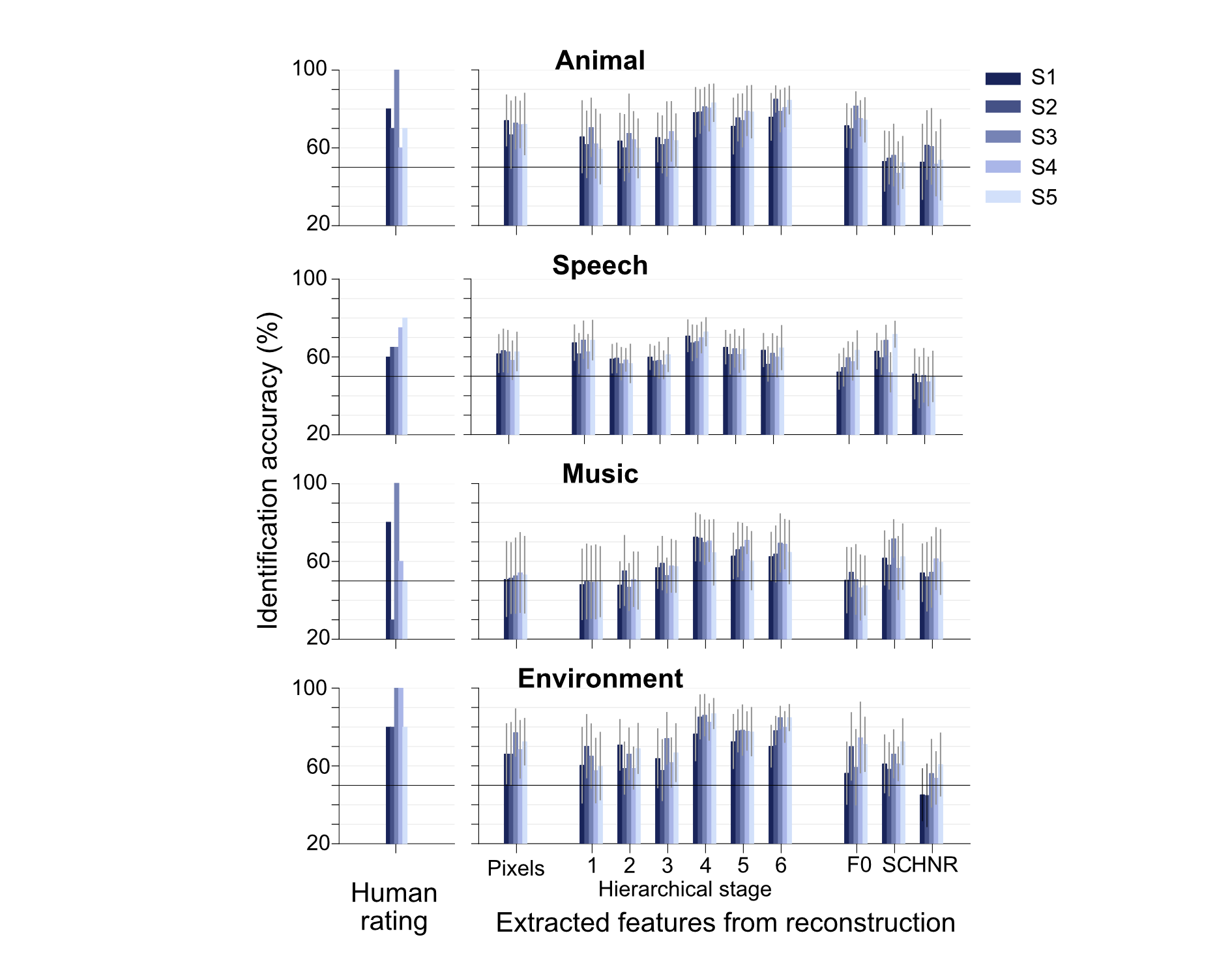

Supplement: S6 Fig — Identification accuracy for reconstructed sounds is presented by category. Each bar represents the mean accuracy across 10 test stimuli for the animal, music and environment categories and 20 test stimuli for the speech category. Error bars indicate the 95% CI, and each color corresponds to an individual subject. Human ratings are generally above 70% for animals and environments, while speech ratings ranged from 60% to 80%, with music showing greater variability. Trends in human ratings closely align with objective evaluations. These results suggest that reconstructed sounds for animals and environments reliably captured category-specific features, enabling accurate identification of true stimuli, whereas reconstructions for speech and music lacked sufficient detail for consistent within-category discrimination. The data underlying this figure are provided in S2 Data. (TIFF) [file pbio.3003293.s006.tiff]

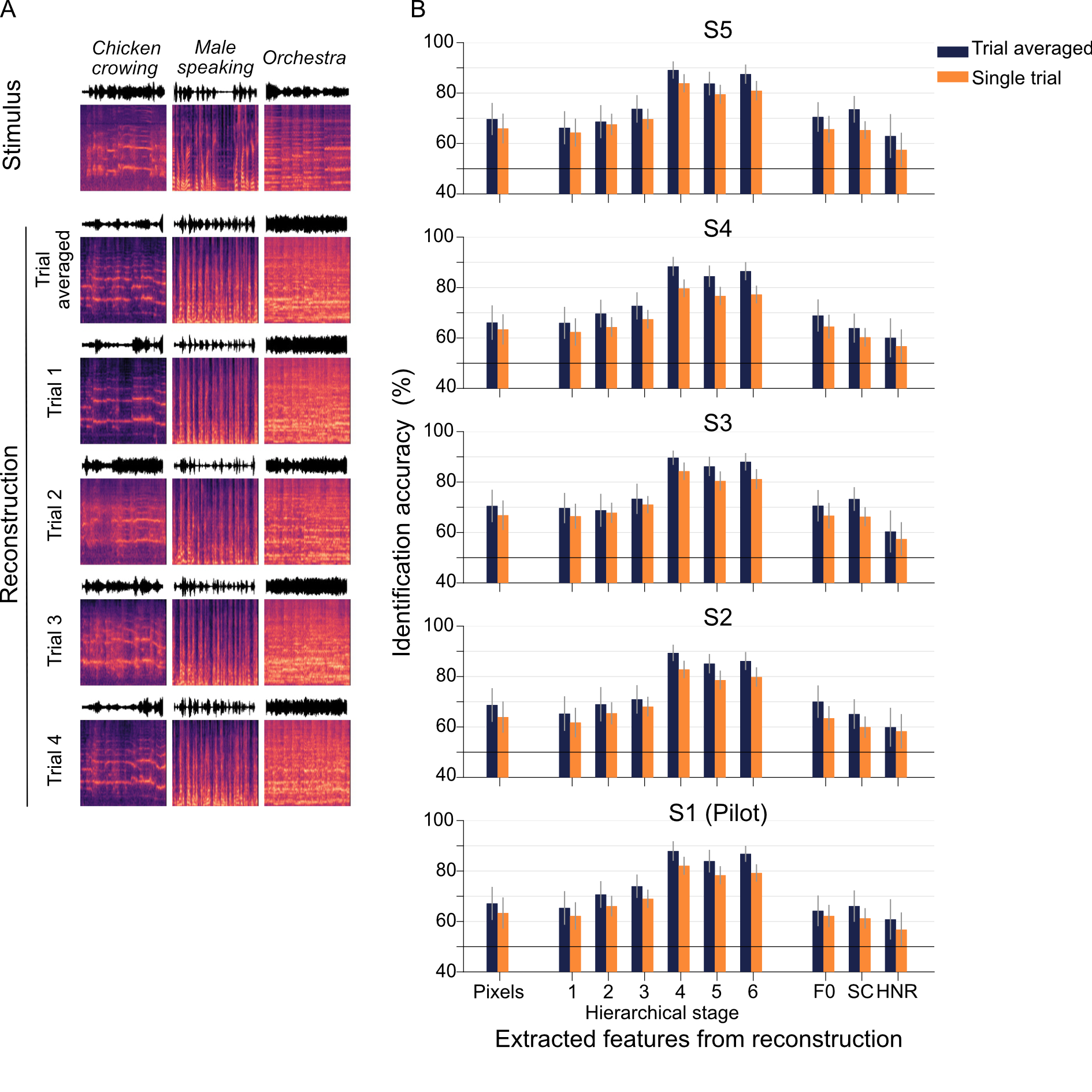

Supplement: S7 Fig — (A) Reconstructed spectrograms (ROI: AC, DNN layer: Conv5; for reconstructed sounds, see https://www.youtube.com/watch?v=exUeKzT0Qfo). The top row shows the original spectrogram of the stimulus sounds. The second row depicts reconstructions based on fMRI samples averaged over eight trials. Rows three to six display reconstructions using single-trial fMRI samples from different trials. (B) Evaluation of reconstructed sounds. Each panel represents the mean identification accuracy for each subject. The dark blue bar shows results from fMRI samples averaged over eight trials, while the orange bar indicates the average accuracy of single-trial reconstructions across eight repetitions. Error bars represent the 95% CI based on 50 data points. Identification accuracy for single-trial reconstructions is approximately 60% for spectrogram and lower hierarchical stages, improving to around 80% in higher hierarchical stages. For acoustic features, the model achieves 60% accuracy for F0 and SC. Despite lower performance compared to trial-averaged reconstructions, these findings confirm the model’s capability to produce reasonable reconstructions from single-trial fMRI samples. The data underlying this figure are provided in S2 Data. (TIFF) [file pbio.3003293.s007.tiff]

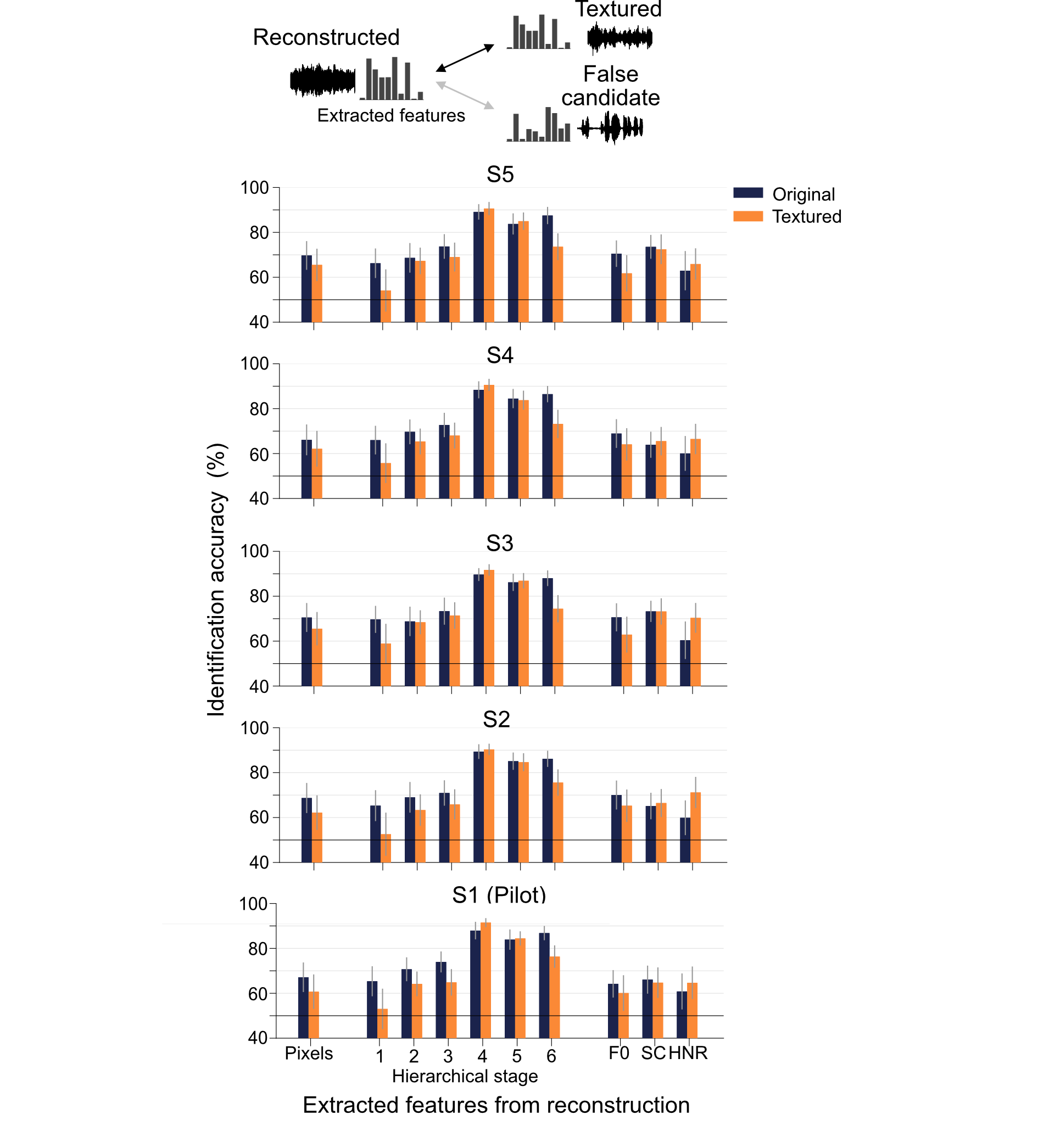

Supplement: S8 Fig — Identification accuracy of reconstructed sounds is shown for each subject when compared against temporally textured true stimuli. Each panel corresponds to an individual subject. Dark blue bars represent accuracy using the original true stimuli, and orange bars indicate accuracy using the textured true stimuli. Error bars denote the 95% CIs based on 50 data points. The data underlying this figure are provided in S2 Data. (TIFF) [file pbio.3003293.s008.tiff]

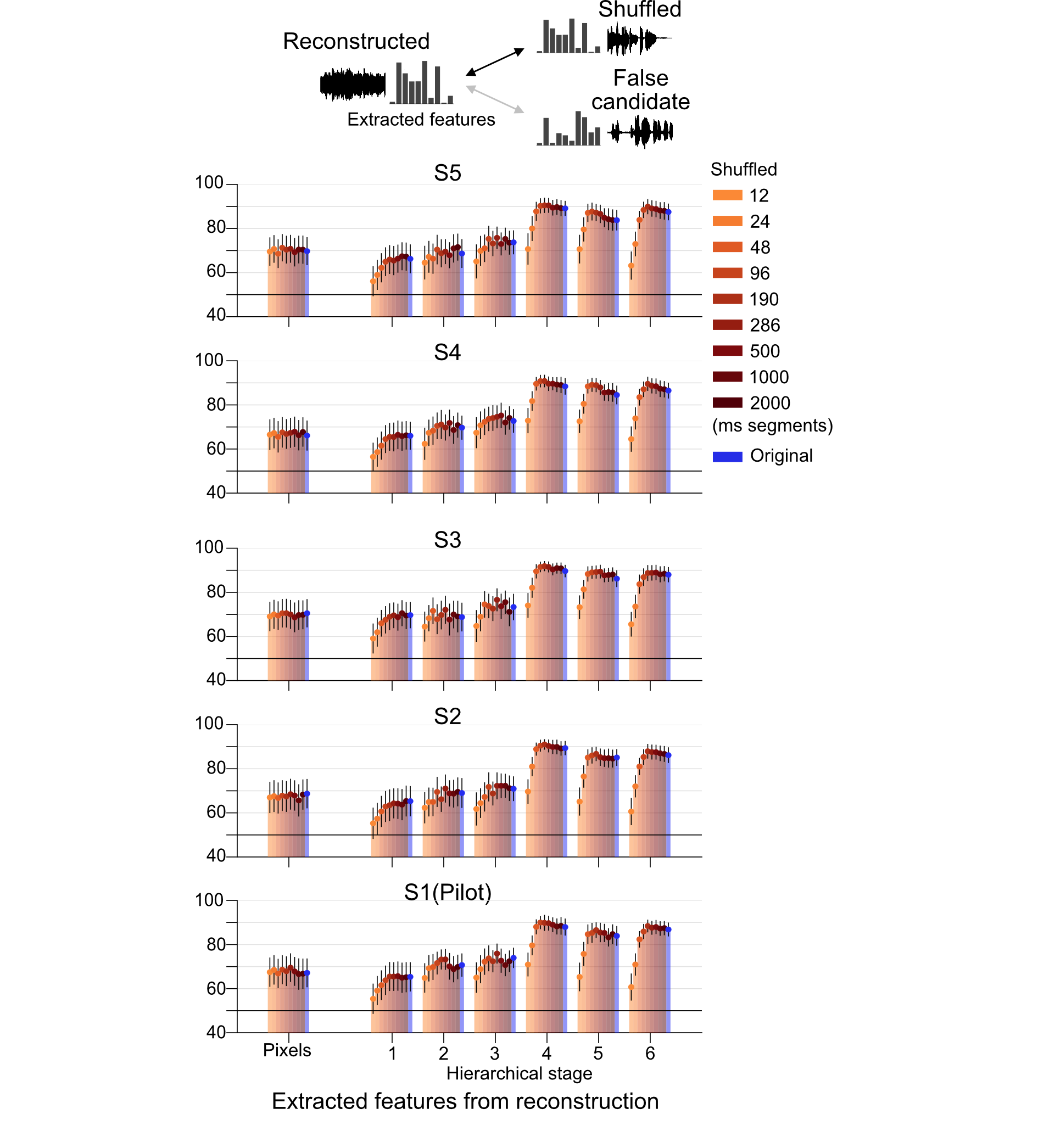

Supplement: S9 Fig — The identification accuracy of reconstructed sounds was assessed using temporally shuffled true stimuli. Each panel corresponds to an individual subject, with each bar representing the mean accuracy for different segment sizes, indicated by varying colors. Error bars denote the 95% CIs based on 50 data points. The data underlying this figure are provided in S2 Data. (TIFF) [file pbio.3003293.s009.tiff]

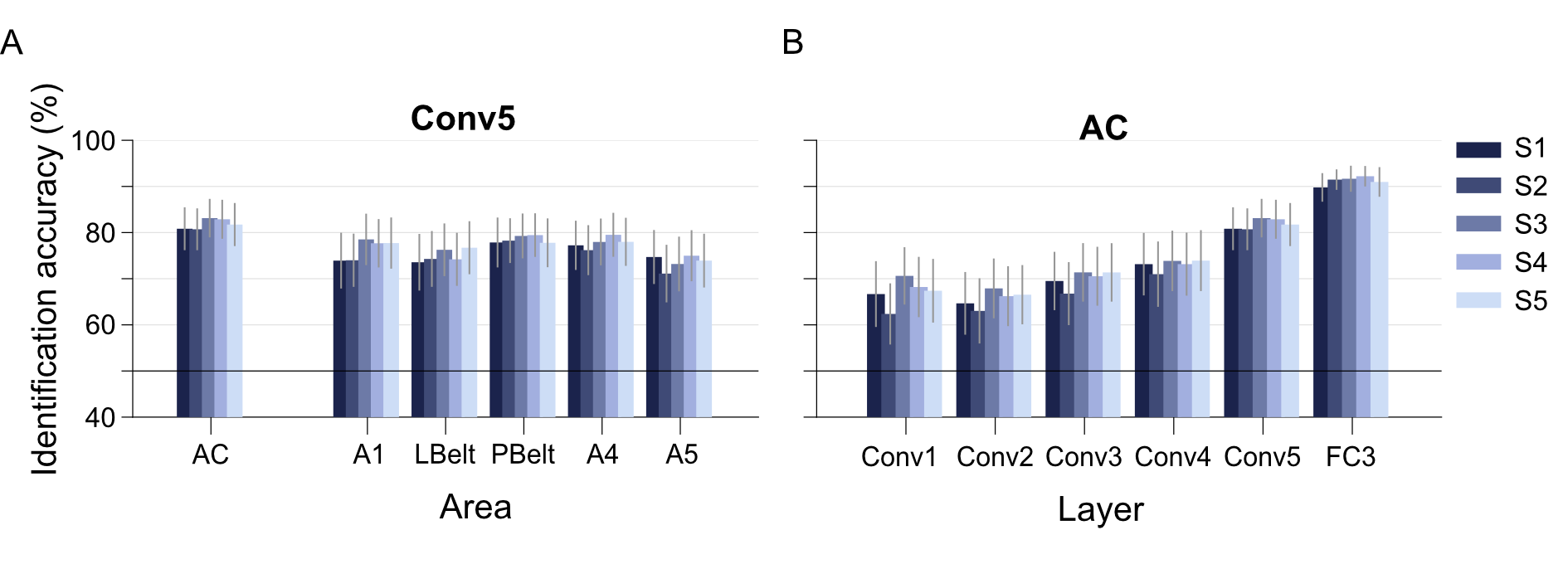

Supplement: S10 Fig — (A) Identification accuracy across auditory areas. Identification accuracy for decoded DNN features from the Conv5 layer is presented for different auditory regions. Each bar represents the average accuracy for individual subjects, with error bars indicating the 95% CI based on 50 data points. Colors correspond to individual subjects. Accuracy consistently exceeds 70% across primary and secondary auditory areas, with minimal variation among regions. These results suggest a distributed auditory processing system rather than a strict hierarchical correspondence between auditory regions. (B) Identification accuracy across DNN layers. Identification accuracy for decoded features from various DNN layers using the AC is shown. Performance improves at higher DNN layers compared to lower layers, demonstrating that hierarchical representations encoded in different DNN layers can be reliably predicted from the AC. The data underlying this figure are provided in S2 Data. (TIFF) [file pbio.3003293.s010.tiff]

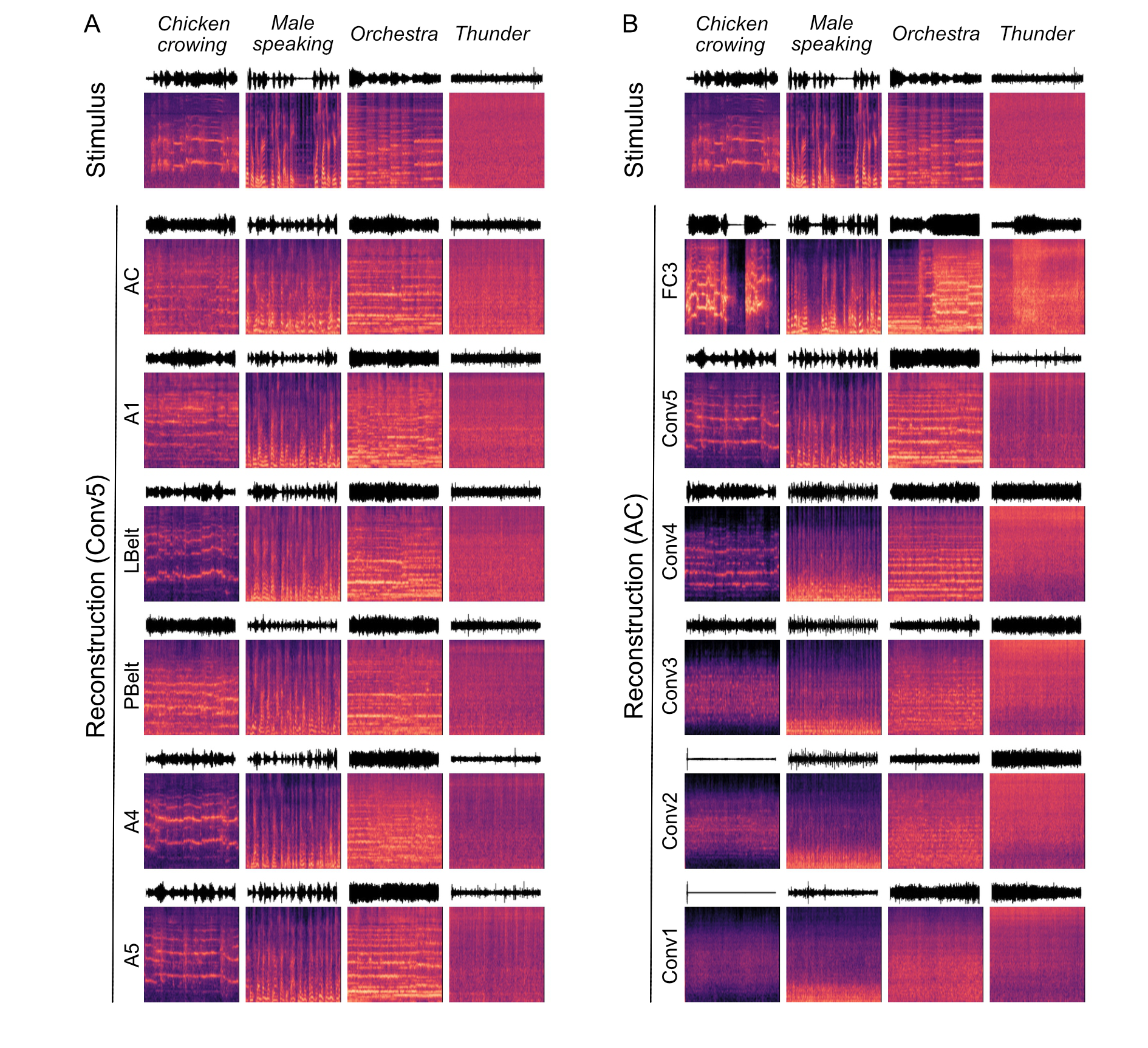

Supplement: S11 Fig — (A) Reconstructed spectrograms from individual ROIs (DNN layer: Conv5; for reconstructed sounds, see https://www.youtube.com/watch?v=qjMg887OOTM). The top row shows the original spectrogram of the presented sound. Rows two through seven display spectrograms reconstructed using fMRI data from individual ROIs, highlighting the contributions of each auditory region. (B) Reconstructed spectrograms using different DNN feature layers (ROI: AC; for reconstructed sounds, see https://www.youtube.com/watch?v=nzR0ibTYmTI). The top row shows the original spectrogram of the presented sound. Rows two through seven represent spectrograms reconstructed using features from different layers of the DNN model, illustrating the influence of hierarchical DNN features on reconstruction quality. (TIFF) [file pbio.3003293.s011.tiff]

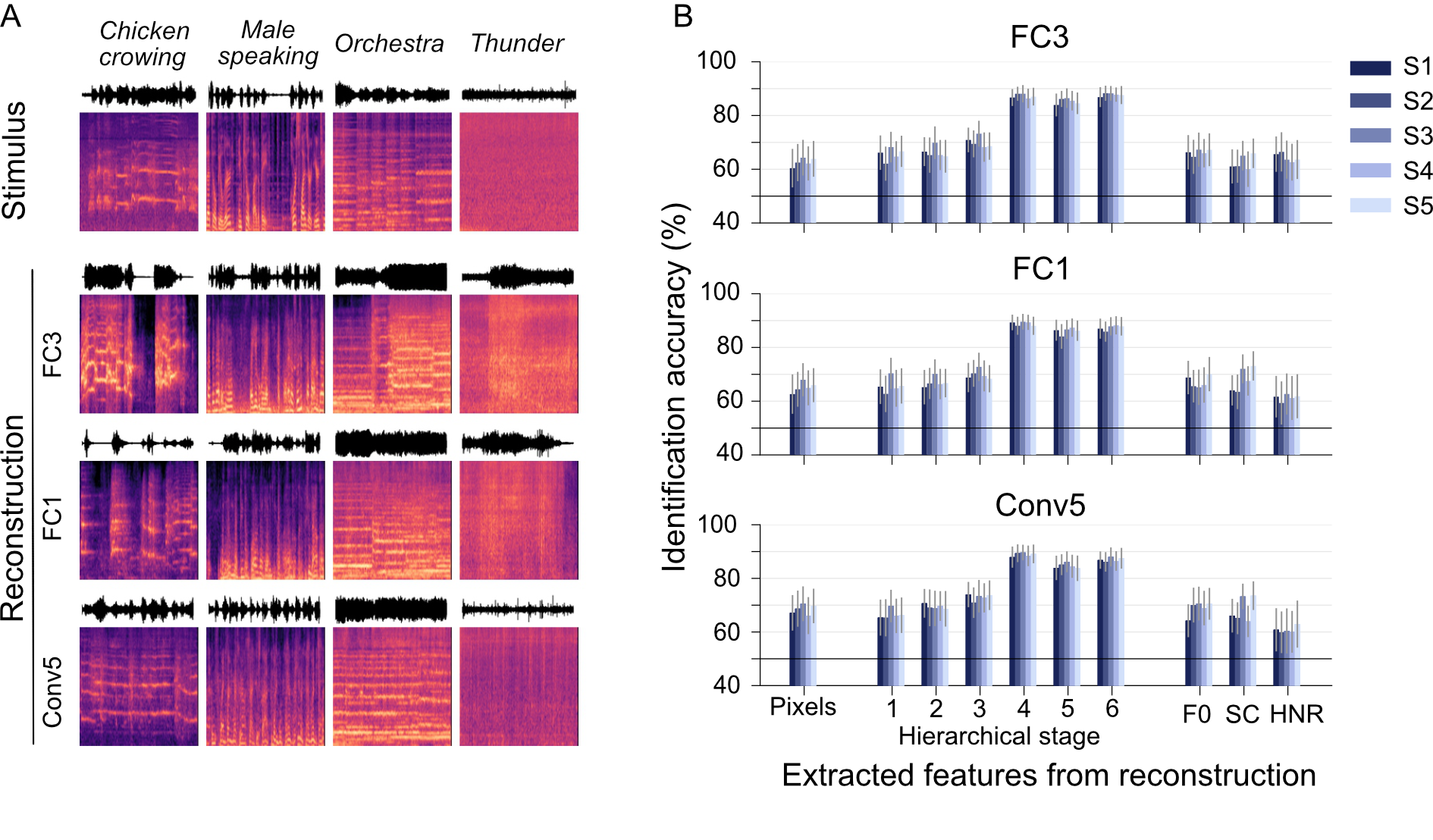

Supplement: S12 Fig — (A) Reconstructed spectrograms generated using decoded features from three DNN layers: the last convolutional layer (Conv5) and two fully connected layers (FC1 and FC3) of the VGGish-ish model. The top row shows the original spectrograms of the stimulus sounds. The following rows display spectrograms reconstructed using features decoded from each layer. (B) Evaluation of reconstructed sounds from Conv5, FC1, and FC3 (ROI: AC). Each bar represents the mean identification accuracy calculated for each subject, with the error bar indicating the 95% CI estimated from 50 data points. While Conv5 tended to show slightly higher performance in pixel-based measures, the overall differences among the three layers were modest and varied across subjects. These results suggest that both convolutional and fully connected layers retain sufficient information for reconstructing perceptual attributes. The data underlying this figure are provided in S2 Data. (TIFF) [file pbio.3003293.s012.tiff]

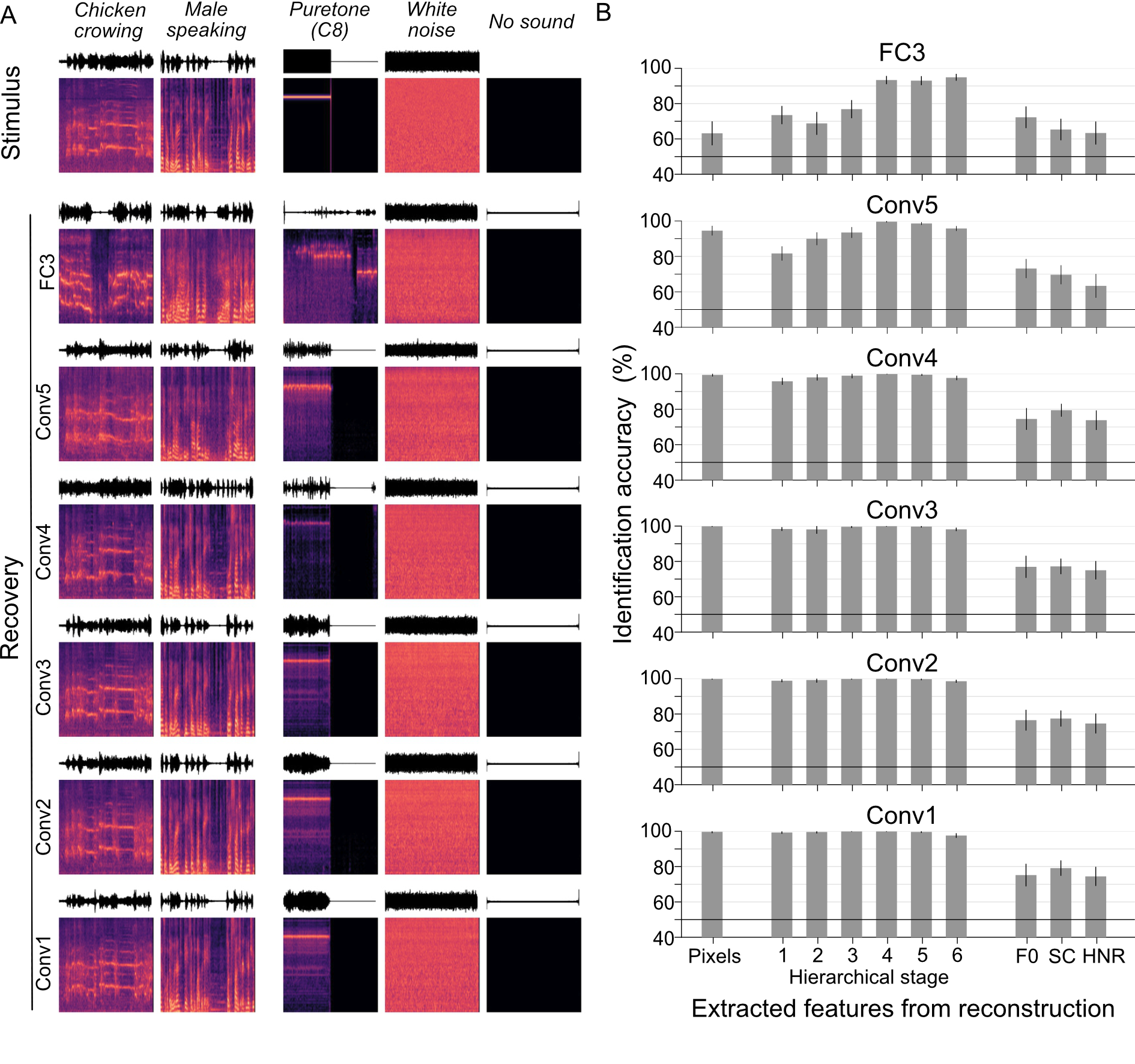

Supplement: S13 Fig — (A) Recovered spectrograms from different DNN layers. The top row shows the original spectrograms of the stimulus sounds, while rows two through seven display spectrograms recovered using true DNN features from different layers. The first and second columns show natural sounds selected from VGGSound, while the third to fifth columns present artificial sounds not included in model training. Recovery from lower DNN layers (Conv1 to Conv3) achieves near-perfect reconstruction of the original spectrograms. Spectrograms from Conv4 and Conv5 show subtle degradation but retain key spectral patterns. Recovery using category-level features from FC3 exhibits more pronounced degradation, particularly in temporal structure, although spectral patterns often remained discernible. Even for artificial sounds, recovery demonstrates high fidelity. (B) Evaluation of recovered sounds from different DNN layers. Bars represent the mean identification accuracy, with error bars indicating the 95% CI based on 50 data points. Recovery from lower layers achieves nearly 100% accuracy. For Conv5, accuracy remains close to 95% in pixel-based evaluations, with a slight decline in lower hierarchical stages and near-perfect recovery (~100%) in higher hierarchical stages. Recovery from FC3 shows significant degradation in pixel-based and lower hierarchical stages but retained over 90% accuracy for higher hierarchical stages. The data underlying this figure are provided in S2 Data. (TIFF) [file pbio.3003293.s013.tiff]

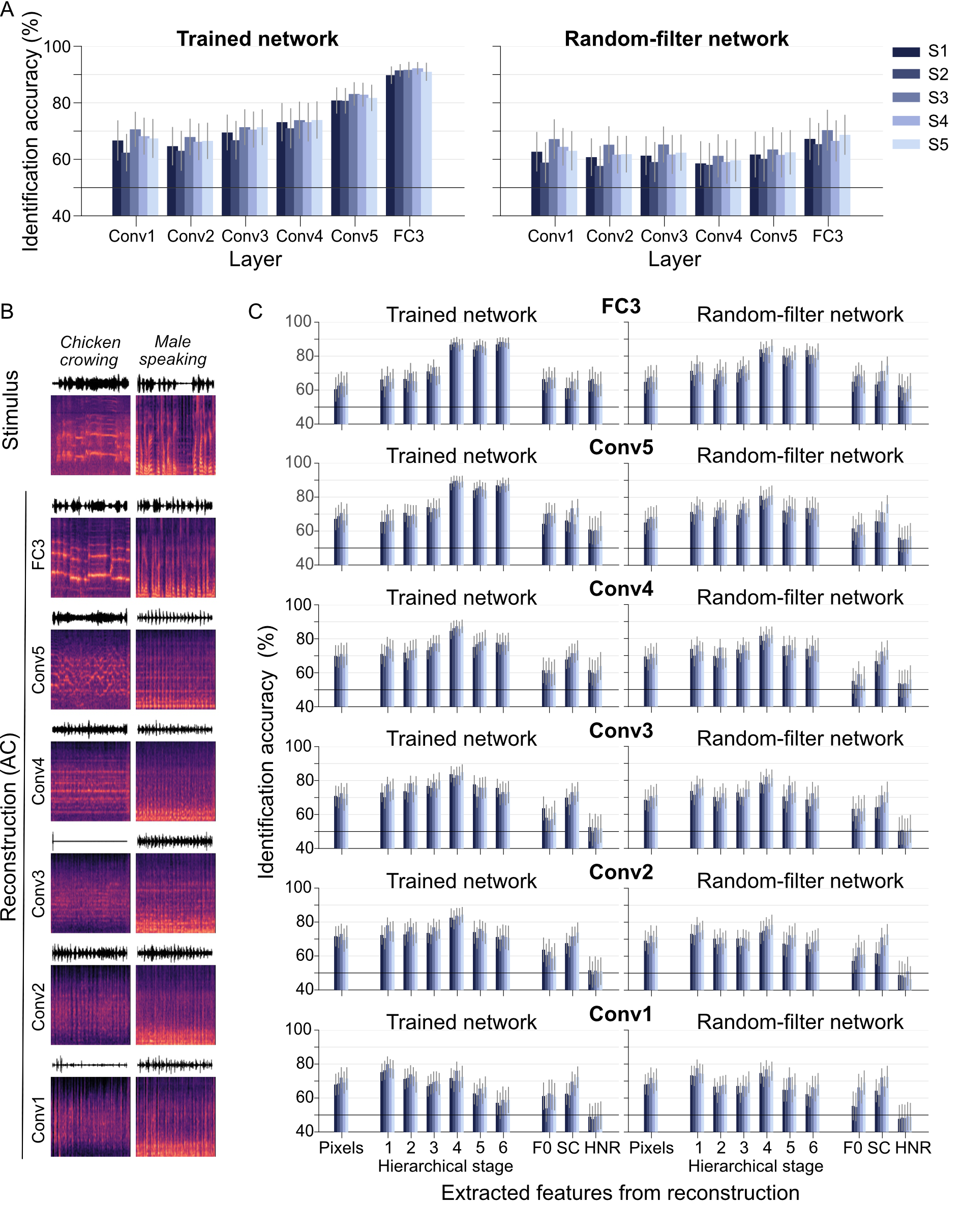

Supplement: S14 Fig — (A) Evaluation of decoded features across DNN layers extracted from an untrained VGGish-ish model (ROI: AC). Decoded features from the trained model show lower performance than those from the untrained model, particularly in Conv5 (our primary decoding target). At lower hierarchical layers, such as Conv1 and Conv2, there are minimal difference between trained and untrained features. However, as layer depth increases, trained DNN features exhibit a gradual performance improvement, while untrained features remain relatively unchanged. (B) Reconstructed spectrograms using different DNN feature layers from an untrained model (see https://www.youtube.com/watch?v=nX6idzcQGMU). The top row presents the original spectrogram of the presented sound, while rows two through seven display reconstructions based on features from different layers of the untrained DNN model. Although these reconstructions reflect the influence of hierarchical DNN features on reconstruction quality, they poorly resemble the original spectrograms. However, reconstructions from the FC3 layer retain some distinct spectrotemporal patterns. (C) Evaluation of reconstructed sounds. The difference between reconstructions using trained and untrained DNN features is marginal. Beyond this, however, a pattern of hierarchical correspondence is observed. At higher hierarchical stages, reconstructions from lower layers show lower performance when using trained DNN features compared to untrained. However, for higher layers, trained DNN features outperform untrained features, showing a shift in performance across layers. These results suggest that while model architecture influenced reconstruction quality, task-optimized DNN features played a role in capturing hierarchical structure and improving perceptual evaluations. The data underlying this figure are provided in S2 Data. (TIFF) [file pbio.3003293.s014.tiff]

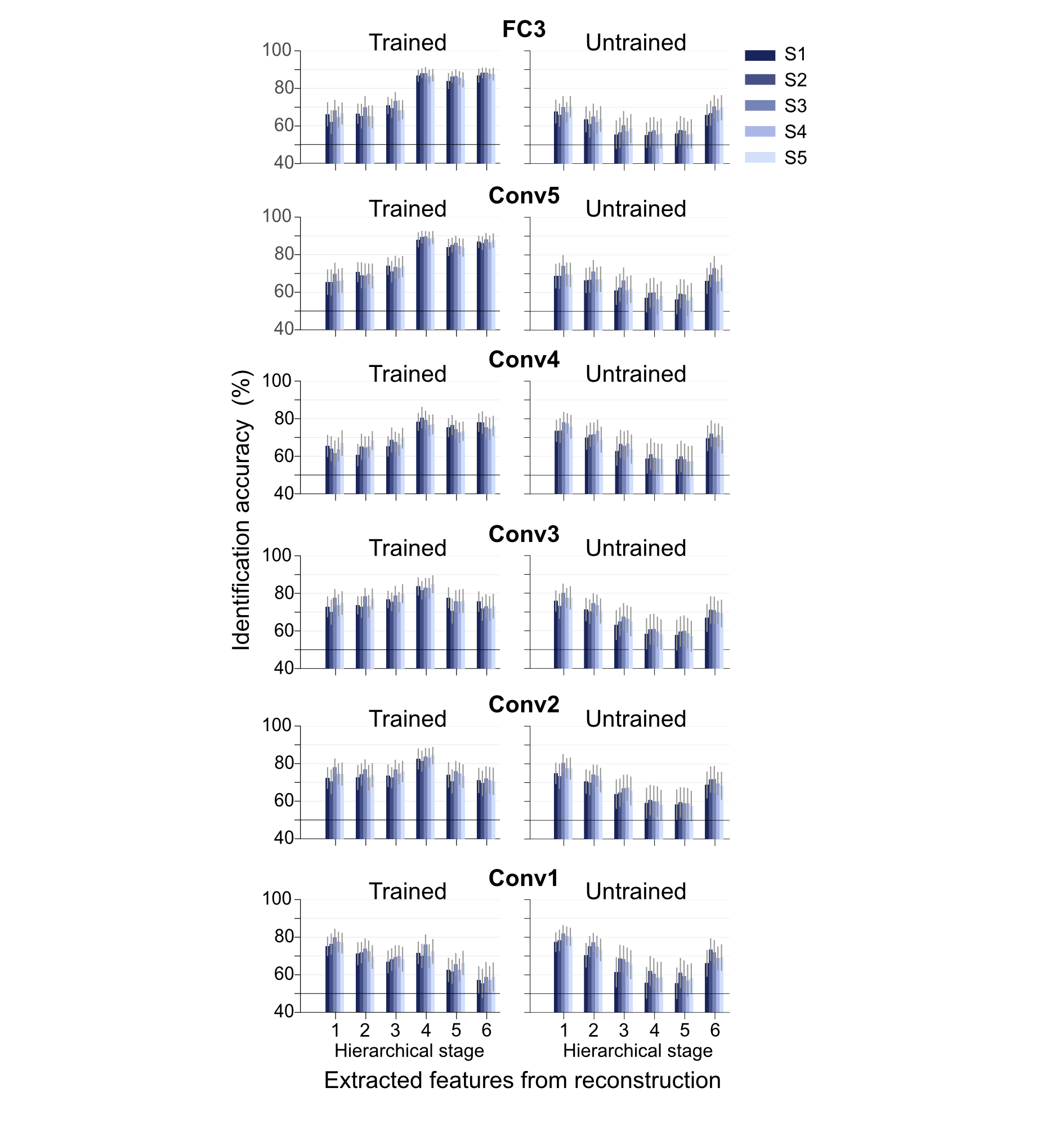

Supplement: S15 Fig — Reconstructed sounds (ROI: AC) were evaluated using a trained Melception model (left panel) and an untrained model with random filter weights (right panel). Each bar represents the mean identification accuracy per subject, with error bars indicating the 95% CI based on 50 data points. Lower hierarchical stages from the untrained model show minimal changes compared to the trained model. However, performance declines across all DNN layers as the representation hierarchy increases. Reconstructions from Conv5 exhibit a nearly 30% drop in performance at the hierarchical stage 4, which aligns most closely with human ratings. These findings underscore the critical role of task-specific optimization in evaluating hierarchical representations and suggest that the observed hierarchical structure in reconstructed sounds is not solely attributed to the model’s architectural characteristics. The data underlying this figure are provided in S2 Data. (TIFF) [file pbio.3003293.s015.tiff]

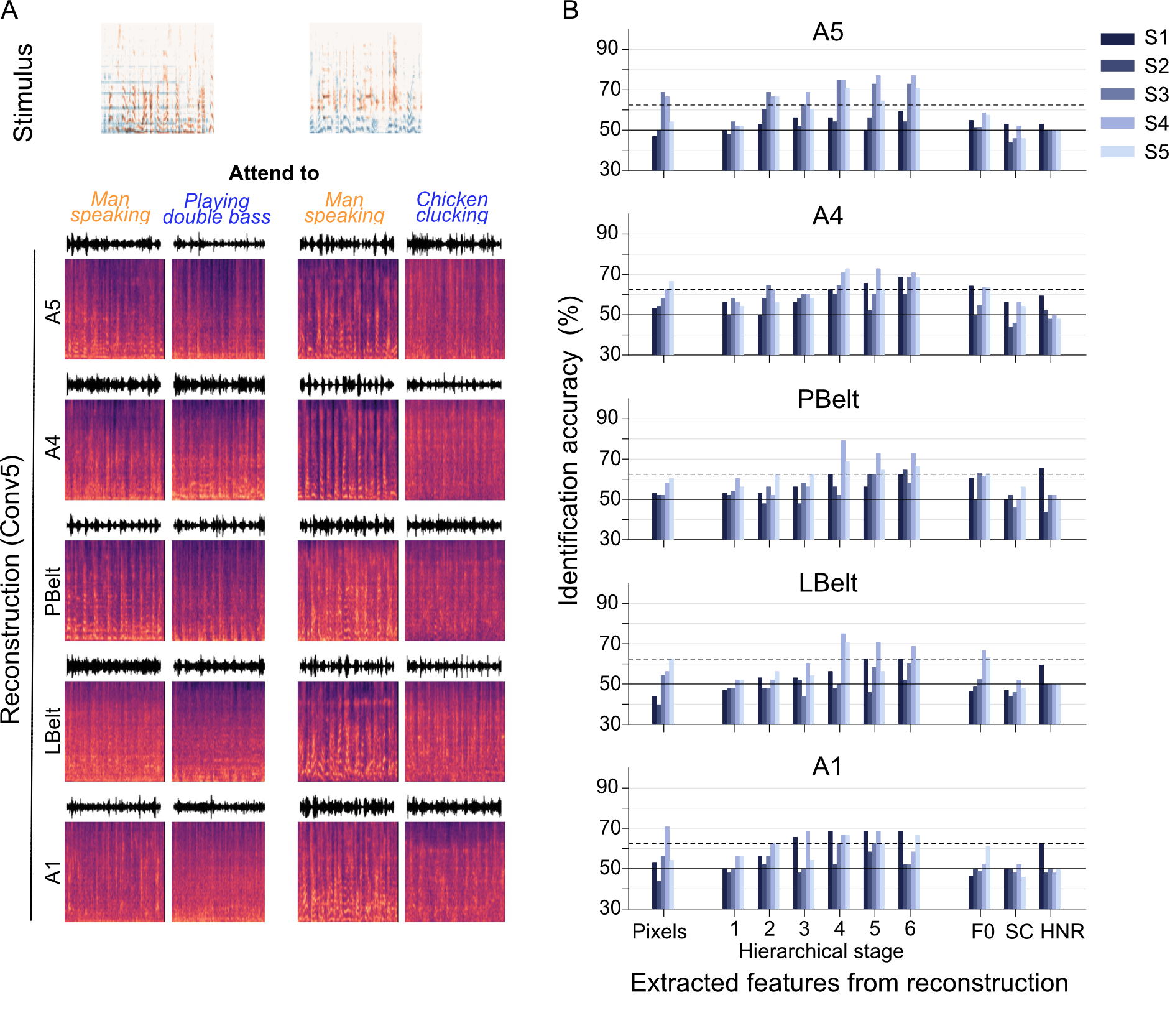

Supplement: S16 Fig — (A) Reconstructed spectrograms from individual ROIs during selective auditory attention tasks (DNN layer: Conv5; for reconstructed sounds, see https://www.youtube.com/watch?v=jJDBq2lx0Aw). The top panel shows the spectrograms of two superimposed sounds presented during the task, where subjects were instructed to focus on one specific sound. The bottom panel displays spectrograms reconstructed from each individual ROI for Subject S4. (B) Identification accuracy of attended sound from individual ROIs. Each panel corresponds to a specific auditory area, with each bar representing the mean identification accuracy for an individual subject based on 48 data points. The dashed line indicates the significance threshold (p < 0.05) as determined by a binomial test. These findings highlight the ability of individual auditory regions to contribute to distinguish attended sounds in the presence of competing auditory stimuli. The data underlying this figure are provided in S2 Data. (TIFF) [file pbio.3003293.s016.tiff]
