# Supplementary material for: Natural sounds can be reconstructed from human neuroimaging data using deep neural network representation
Source: PLoS Biol. 2025 Jul 23;23(7):e3003293. doi: 10.1371/journal.pbio.3003293 (PMC12313072; doi:10.1371/journal.pbio.3003293)
Supplement: Attachment — Submitted filename: Response_250425.docx [file pbio.3003293.s030.docx]

Responses to reviewers: “Sound reconstruction from human brain activity via a generative model with hierarchical auditory features”

We sincerely thank the editor and reviewers for their insightful feedback, which significantly improved the clarity and quality of our manuscript. We have carefully reviewed each comment and made appropriate revisions to address all issues raised. Below, we provide detailed, point-by-point responses.

(Reviewers’ comments in italics)

**Reviewer #1**

(1)

*The authors present an fMRI dataset collected on a wide range of natural sounds with the goal of decoding the auditory response from the fMRI activity. The decoding relies on a few stages. First, features of a CNN (or control representation such as a spectrogram or spectrotemporal filters) are reconstructed using L2 regularized regression. Second, these features are turned into a spectrogram using a generative model, specifically a version of "SpecVQGAN." To evaluate the reconstructed spectrograms, the authors used other feature sets, for instance, representations of the "Melception" network or "auditory" features such as the F0 or Spectral Centroid. For qualitative evaluation, a vocoder was used to turn the spectrograms into audio. The paper claims that CNN features have better decodability from the fMRI responses and that reconstructions from these features have perceptual qualities similar to the original audio. Although the paper uses an interesting fMRI dataset and is trying to tackle the difficult problem of reconstructing audio from fMRI, the results fell flat for me, and I believe that a significant amount of work (experiments, analysis, and clarity of writing) would be required to raise this to an appropriate level of publication.*

**Response:** We thank the reviewer for their thorough review and insightful feedback. Below, we provide detailed responses to each of the comments, highlighting the revisions made to address the concerns raised.

(2)

*Major:*

*Overall, I find the modeling efforts a bit difficult to follow (even as someone who regularly works with these types of models). There are many different components that are pasted together, which makes it difficult to understand what each individual component is doing. The authors have run some ablation experiments to try and show the contribution of each, but I believe that the wording and explanation in the paper would significantly benefit from clear, high-level conclusions about what each part of the model contributes. This seems especially important for a journal like PLOS Biology, which should be accessible to non-specialists.*

**Response:** To improve clarity and accessibility for a broad readership, we have substantially revised the manuscript to explicitly describe the role and contribution of each model component. The updated text introduces a high-level overview in both the Introduction (lines 123–128) and Results sections (lines 235–252), supported by revised schematics (Fig 4A-B) and supplemental materials (S1 Fig and S5 Fig).

We now describe our reconstruction pipeline as a multi-stage system with three interconnected components: (1) a codebook system (SpecVQGAN), (2) an audio transformer, and (3) a vocoder. Each plays a distinct role in the transformation from fMRI-decoded DNN features to reconstructed sounds:

- SpecVQGAN codebook compresses spectrograms into a compact set of discrete indices. It learns a dictionary of spectrotemporal patterns from training data and enables efficient encoding of sound structure by mapping spectrogram patches to learned code entries (S1 Fig).
- The audio transformer autoregressively predicts these codebook indices from brain-decoded DNN features. It learns the temporal and spectral dependencies needed to generate a coherent code sequence representing the spectrogram (Fig 4A).
- The vocoder (MelGAN) converts the generated spectrogram back into an audible waveform, completing the sound reconstruction.

We further clarified each component’s contribution by conducting ablation analyses using alternate reconstruction pipelines: one that bypasses the transformer and directly predicts the codebook, and another that replaces the codebook with pixel-level optimization (S5 Fig). These experiments show that both the audio transformer and the learned codebook are essential to maintain the perceptual structure and fidelity of the reconstructed sounds.

We hope this clearer description helps readers grasp the structure and function of our generative reconstruction system.

(3)

*In the introduction and motivation of the paper, the authors extensively highlight how the temporal resolution of fMRI has limited its use for decoding, which at least leads the author to believe that the proposed method will capture temporal processing. However, the authors' evaluations don't sufficiently address whether their decoded stimuli are more temporally similar to the originals, and in fact when I listened to the examples it seems like the sounds do not preserve temporal aspects at all (everything sounds very scrambled or texturized). To make a claim about extracting temporal components from the fMRI signal, the authors would need a quantitative analysis that specifically addresses what temporal aspects are preserved/not preserved. Unfortunately the acoustic evaluations all rely on representations that are collapsed across time. Because of this, I do not believe one can claim that the reconstructions capture "detailed acoustic" characteristics (Line 289) or that the reconstructed sound "approximately preserv[es] the content and quality of the original stimulus" (507-508). These sections should be modified.*

**Response:** We agree that the coarse temporal resolution of fMRI raises critical questions regarding the extent to which temporal structure can be preserved in reconstructed sounds. While our initial acoustic evaluations focused on features averaged across time, we have now performed additional analyses to directly assess the degree of temporal preservation in the reconstructions.

To address this, we conducted two identification analyses using temporally perturbed stimuli (see revised Results, “Temporal structure of reconstructed sounds,” lines 352–392). These analyses quantitatively test whether the reconstructed sounds reflect short- or long-term temporal dynamics encoded in brain activity.

First, we examined whether reconstructions merely resembled time-averaged textures of the original sounds by comparing them to sound textures synthesized from statistical averages (Fig 5A–C). Results showed that performance declined when comparing reconstructions to textured versions at low-level feature stages, suggesting that the reconstructions retained temporal patterns beyond static spectral statistics. In contrast, performance remained stable at mid-level feature stages (*e.g.*, hierarchical stage 4), indicating perceptual resemblance even when temporal detail was smoothed.

Second, we assessed sensitivity to temporal scrambling by evaluating identification accuracy across varying time window sizes (Fig 5D–F). We compared reconstructed sounds to true stimuli whose spectrograms were temporally shuffled in segments ranging from 12 ms to 2 s. Identification performance declined sharply for short segments but was preserved for windows >100 ms, suggesting that the reconstructions retained short-term temporal structure (~100 ms) but lacked longer-sequence fidelity.

These results clarify that although the model does not reconstruct precise temporal sequences (*e.g.,* speech rhythm or melody), it preserves temporally local sound features that distinguish between stimuli. Accordingly, we have revised claims in the manuscript to emphasize that they retain short-term spectral and perceptual characteristics while exhibiting limitations in long-term temporal fidelity.

(4)

*Related to the temporal resolution, it is possible that a large amount of the "hierarchy" observed (ie Figure 6B) is due to the receptive field sizes of the models. Control experiments for this should be conducted, for instance, using models with random rather than learned weights.*

**Response:** We agree that to attribute the observed hierarchy in Figure 6B to task-specific learning rather than architectural factors such as receptive field size, appropriate control analyses are required. In response, we performed additional control experiments using models with random (untrained) weights to disentangle the effects of receptive field structure from learned representations. These results are now described in the revised manuscript as summarized below.

First, we assessed feature decoding performance across DNN layers using an untrained version of the VGGish-ish model (S14A Fig). While decoding performance from lower layers (e.g., Conv1, Conv2) remained comparable between trained and untrained models, deeper layers (e.g., Conv5) showed a marked drop in accuracy when using untrained features. This suggests that the hierarchical decoding performance in trained models cannot be fully explained by differences in receptive field size, but instead arises from learned auditory representations (Fig 3C–D and lines 197–199).

Second, we examined how using untrained DNN features affects the reconstruction quality. As shown in S14 Fig and S9 Movie, spectrograms reconstructed from untrained features failed to resemble the original sounds, especially at higher layers. This result further confirms that the observed hierarchy in reconstruction fidelity is not an artifact of architectural depth or receptive field size alone, but depends critically on task-specific feature learning (lines 475–481).

Finally, to test whether the evaluation metrics themselves reflect learned representations, we used an untrained Melception model to evaluate the reconstructed sounds (S15 Fig). At hierarchical stage 4—previously shown to align well with human perception—reconstruction performance dropped by nearly 30% when evaluated with the untrained model. This indicates that task optimization is also essential in the evaluation model to capture meaningful hierarchical relationships in the reconstructions (lines 483–487).

We believe these three complementary control analyses—at the levels of decoding, reconstruction, and evaluation—effectively address the concern that the observed hierarchy might stem from receptive field properties alone. We have updated the relevant Results sections accordingly to clarify the implications of these findings.

(5)

*The authors refer to the test dataset as having both 50 and 150 samples (for instance, lines 165-170). After reading the methods in detail, I understood that this was not a typo and in fact the 150 sounds are from 3x crops of each of the 50 sounds. This (and the non-independence of the 150 points) should be made clear in the main text, and references to the "test" dataset sample size should be made consistent throughout the paper. Currently, the description on lines 134-136 does not adequately convey the 50 vs. 150 samples. Additionally, the 4s crops are not depicted in the methods schematics.*

**Response:** We thank the reviewer for pointing out the ambiguity in our description of the test dataset sample size. We agree that the distinction between the number of stimuli (50) and the number of cropped data samples (150) should be made explicit, especially given the non-independence introduced by temporal overlap.

To clarify, we revised the main text (lines 159–172) to explicitly state that each 8-s test stimulus was divided into three overlapping 4-s segments, yielding a total of 150 data samples. These samples were used for decoding and reconstruction analyses. However, since the segments originate from the same original stimulus and share temporal overlap, they are not statistically independent.

To ensure proper statistical evaluation, we first computed results for each of the 150 segments and then averaged across the three segments of each stimulus to obtain 50 independent test points, which were used for calculating statistics such as confidence intervals (CI). This approach is now clearly stated in both the Results and Materials & Methods sections (lines 740–750 and lines lines 987–996).

We also updated the schematic in Fig 2D to visually depict the 4-s segmentation of 8-s stimuli, aligning the visual presentation with the revised text.

(6)

*Also in relation to the above, why were the 4s crops determined? This seems rather ad-hoc, makes the methods difficult to follow, and complicates the statistics (due to the non-independence, as detailed in lines 897-902. Is it possible to evaluate the test datasets with a fixed crop window in all experiments to simplify the analyses?*

**Response:** We appreciate the reviewer’s comment and fully agree that clarity in our data sampling strategy is essential.

The 4-s crop windows were introduced as a data augmentation strategy to increase the number of training and test samples per stimulus. Given the relatively small number of available trials, dividing each 8-s stimulus into three overlapping 4-s segments allowed us to expand the training set threefold without requiring additional data collection. This approach slightly improved decoding performance compared to using a single fixed crop window in our preliminary analysis: Profile correlation improved from 0.435 (fixed crop window) to 0.460 (proposed). We agree that overlapping crops complicate statistical assumptions due to their non-independence. To address this, we averaged the predictions from the three segments of each stimulus and conducted statistical analyses on the resulting 50 independent test points, as clarified in the revised Results (lines 159–172) and Methods sections (lines 987–996). We hope this clarifies the motivation behind our design choice.

(7)

*The "identification analysis" should be better described in the main text. How are the "true" and "false" candidates (Fig 3A) compared to each other? In the methods (line 877) it states that the "correlation coefficient" was calculated between the reconstruction and candidate stimuli, but is this is Pearson Correlation coefficient? Would the results change if one used a different similarity measure with different biases (Spearman correlation, L2 distance, etc)? The precise evaluation should be explained in the main text, as the exact similarity measure used could bias the results as Pearson correlations are very sensitive to outliers (which could drive the "good" models up or drive the "bad" models down). Additionally, the main results should be replicated with a different type of similarity measure to verify that this is not dependent on the measure being used.*

**Response:** We have revised the main text (lines 207–216) to more clearly describe our identification analysis procedure and the similarity measure used. Specifically, we now state that Pearson correlation was used as the default similarity metric to compare decoded feature vectors against those of the true and false candidate stimuli. For each decoded feature, we computed the Pearson correlation with the feature vectors of the 49 test stimuli, and a sample was considered correctly identified if the true stimulus yielded the highest correlation.

We also conducted an additional analysis using Spearman correlation, showing similar results (R1 Fig). Thus, in our manuscript we show the results with Pearson correlation as representative ones as indicated in "Materials & Methods: Feature decoding analysis" (Fig 3D and lines 870–872).

**R1 Fig. Comparison of decoding performance between Pearson and Spearman correlation.** (A) Identification accuracy for decoded auditory features. Each panel illustrates the mean identification accuracy for individual subjects. The dark blue bars represent evaluations based on Pearson correlation, while the orange bars denote accuracy using Spearman correlation. Each bar reflects the mean accuracy across 50 data points, with error bars showing the 95% confidence interval (CI). DNN units exhibiting negligible response variability across the training dataset stimuli (standard deviation < 0.000001), which can adversely affect rank calculations due to artifacts, were excluded from the Spearman correlation analysis. (B) Evaluation of reconstructed sound. Identification accuracy for reconstructed sounds was evaluated using both correlation measures, illustrating the robustness of trends across features and representations.

(8)

*One of the biggest flaws in the paper is that there are no quantitative evaluations of human responses to the synthesized stimuli, neither behaviorally nor by collecting new fMRI data for the decoded stimuli. The authors make qualitative claims about the sounds, but these should be backed up by experimental data. For instance, what is the human "identification" accuracy (i.e. a matching task between the original and reconstructed for each model)? This seems more relevant than the scores reported from other computational models and acoustic measures as biases from those models/measures themselves may differ from human perception. Collecting this data may be time-consuming, but I believe it is critical for the interpretation of the study. For instance, in lines 256-265 the authors offer an interpretation of some of the synthetic stimuli, but observations should be quantified  (especially when they are reported as results). Other cases are lines 45, 326, 381, 429, and others where statements are made about "perceptual performance" or "perceptual qualities"; however, there are no perceptual experiments, so this phrasing should be removed.*

**Response:** We fully agree that perceptual judgments by human listeners are critical to substantiate claims regarding the quality of reconstructed sounds. To address this limitation, we conducted a subjective behavioral evaluation in which human raters performed a pairwise identification task. Each trial involved listening to a reconstructed sound alongside two candidate stimuli—one corresponding to the true stimulus and the other a randomly selected lure. Participants judged which candidate was more similar to the reconstruction. Reconstruction accuracy was defined as the proportion of trials in which the true stimulus was correctly identified.

As shown in Fig 4D, identification accuracy was consistently higher than chance across all five subjects (89.2%, 83.6%, 90.4%, 86.8%, and 90.4%, respectively; 95% CI included). Category-wise analyses revealed high accuracies for Animal and Environment categories (>85%) and slightly more variable but still robust results for Speech and Music.

We have incorporated these results into the revised manuscript (lines 279–296 and S2 Fig) and detailed the evaluation procedure in the "Materials & Methods: Identification by human raters" section.

(9)

*Overall, statistics not present in the paper, and rough estimates of values are given instead. Some places where comparisons should be backed up with statistics and/or measures of the exact value and the error range are lines 175,177,183,185, 279,285,287, 361, etc (more, but too many to list).*

**Response:** In response, we have revised the manuscript to include exact numerical values along with their 95% CI wherever key comparisons or performance metrics are reported.

For examples, we added CI values for:

- Decoding accuracy across features and subjects (*e.g*., lines 218–223),
- Human identification performance (lines 290–292),
- Quantitative evaluations (lines 310–317).

(10)

*Figure 3C/SI Fig: This figure is uninformative in terms of results (it perhaps explains what was done, but this is not quantitative). There is a missing number for an SI fig on Line 177, which I presume is SI Fig 1. However, SI Fig 1 shows that Spectrogram model has *higher* correlation (once again, is this Pearson or Spearman, as this will significantly bias the results?) than the DNN based model. **This figure should be in the main text and addressed more directly, and error bars should be included, as it goes against the authors main interpretation**.*

**Response:** We agree that the profile correlation results should be presented more transparently and quantitatively, especially as they reveal subtle differences across feature types. To address this, we have made the following revisions:

1. We updated Fig 3C in the main text to include full quantitative comparisons between spectrogram, modulation, and DNN features, along with subject-wise error bars (95% CI).
2. We clarified in the text (lines 203–205) that profile correlation alone does not reflect discriminability, and that DNN features—despite lower correlation—consistently outperform other features in identification accuracy, which is more sensitive to stimulus-specific variation. This helps reconcile the apparent contradiction and aligns with the reviewer’s concern.
3. To address potential metric bias, we replicated the profile correlation analysis using Spearman correlation, which is more robust to outliers. As shown in R2 Fig, the overall trends remained consistent: spectrogram features yielded higher correlation values, but DNN features still led in identification accuracy. These results further confirm that our conclusions are not dependent on the choice of similarity metric. This point has been noted in the Fig 1 legend and in lines 870–872 of the Methods section.

**R2 Fig. Comparison of decoding performance using Pearson and Spearman correlations.** Mean profile correlation for decoded auditory features across all units, evaluated for each subject. The dark blue bars represent results based on Pearson correlation, while the orange bars show results using Spearman correlation. Each bar indicates the average profile correlation across all units for the respective metric, with error bars representing the 95% CI.

(11)

*One explanation for the difference between the identification accuracy and the profile correlation is that some of the models (i.e., the spectrogram model) may not have much diversity in the feature output and/or that this diversity interacts with the measure being used (once again, Pearson correlation is very susceptible to outliers). I believe it should be possible to compute a "baseline" profile correlation value for each model by computing the correlation between random pairs of natural stimuli, and the measured decoded feature profile correlation can be compared to the random baseline for each model. Due to this possible explanation, I'm not sure that the explanation given for the discrepancy in lines 190-196 is completely correct.*

**Response:** We agree that differences in feature diversity and unit count across models, as well as susceptibility to outliers in the correlation metric, may contribute to the discrepancy between profile correlation and identification accuracy. To assess this issue, we followed the reviewer’s suggestion and computed baseline profile correlations by calculating correlations between random pairs of natural stimuli. The resulting values were near zero (see R3 Fig), indicating that the measured profile correlations are meaningful and not due to statistical bias. Accordingly, we revised the manuscript (lines 199–203) to explicitly state that these baseline comparisons were performed and that the low baseline values support the interpretation of profile correlation as a valid indicator of decoding quality.

Nonetheless, direct comparisons of profile correlation across auditory features remain difficult due to inherent differences in feature distributions and unit dimensionalities. In contrast, identification accuracy—which evaluates whether decoded features can distinguish the true stimulus from alternatives—offers a more interpretable and robust metric for comparing feature types.

Accordingly, we report both profile correlation and identification accuracy results in the manuscript. While the profile correlation demonstrates that all auditory features exhibit positive correlations with the true features (lines 196–197), comparative evaluations across feature types were based on identification accuracy, which more directly reflects the discriminability of decoded features.

**R3 Fig. Baseline profile correlation for decoded features.** Baseline profile correlation for each model was calculated by measuring the correlation between random pairs of natural stimuli. Each panel shows the mean profile correlation for individual subjects. The dark blue bars represent evaluations based on the correct pairs (decoded features vs. true stimuli), while the orange bars show the baseline correlation derived from random pairs. Each bar indicates the mean profile correlation based on Pearson correlation, averaged across all units, with error bars representing the 95% CI.

(12)

*In the section where different layers are used for decoding, it becomes difficult to keep track of what features are being decoded vs. what features are used in the analyses. For instance, in SI Fig 7, it is unclear what type of feature is being used for the correlation and identification analyses. Are these the average across the melception features, the acoustic features, and the spectrogram features? Each of these types of features might tell you different things, so naively it seems inappropriate to collapse across them. Generally though, it should be made clear what is being evaluated and tested, as this is just one case of many in the paper which would benefit from clarity in the writing.*

**Response:** We agree that the original presentation, particularly in sections involving decoding from different layers, may have lacked clarity regarding which feature types were used for evaluation or decoding target. In the revised manuscript, we addressed this by streamlining analyses that were not directly relevant to the main reconstruction framework. Specifically, we removed feature decoding results involving combinations of DNN layers and auditory ROIs, as these analyses may have led to confusion regarding the decoding targets.

In the remaining analyses, we ensured that the evaluated feature types are explicitly and consistently stated. Importantly, we did not average across different feature types (*e.g.,* Melception, acoustic, or spectrogram features), as each provides complementary but distinct information about the reconstructed sounds. This revision improves interpretability and ensures that each analysis is aligned with a clearly defined evaluation metric.

(13)

*Is the sound-reconstruction decoder fidelity for each layer in Figure 6B of equal fidelity? This is a critical detail, as it is possible that the results in Figure 6 are due to *worse* reconstructions for the earlier layers, making it appear that something is "hierarchical" when it is just due to a worse decoder.*

**Response:** We fully agree that differences in reconstruction fidelity across layers could potentially confound the interpretation of the hierarchical effects presented in Figure 6B. If earlier layers yielded inherently poorer reconstructions due to limitations in the audio generator, the observed trends might reflect these artifacts rather than genuine differences in the decoded features.

To address this concern, we conducted a control analysis evaluating the audio generator’s capacity to reconstruct sounds from true DNN features at each layer (S13 Fig). This analysis isolates the generator’s performance from that of the brain decoder. The results showed that spectrograms recovered from lower-layer features closely resembled the original sounds, with only minor degradation in higher layers such as Conv4 and Conv5.

These findings suggest that the differences in reconstruction quality observed across layers primarily arise from the decoded features rather than limitations of the audio generator itself. We have clarified this point in the revised manuscript (lines 468–473) to ensure accurate interpretation of the hierarchical analyses.

(14)

*Overall though, I don't quite understand the logic behind evaluating hierarchical features using a decoding model. For instance, if one had access to all of the responses in the auditory nerve, one could presumably obtain a perfect reconstruction, and all of the decoded features would have high fidelity. Lines 396-401 should be revised to make the logic more clear.  Additionally, the claims made in these lines should be backed up with statistical tests (as I'm not sure that the trends highlighted as significant).*

**Response:** We appreciate the opportunity to clarify the rationale underlying our methodology and refine the interpretation of our findings. In decoding hierarchical DNN features, our objective was to determine whether specific auditory brain regions represent information in an explicit manner that can be extracted through linear transformations alone. We deliberately employed a linear decoder to test the explicit availability of information within each region. This approach aligns with established frameworks in both vision and auditory neuroscience, which infer representational content based on what can be linearly decoded from localized neural activity patterns. Accordingly, we have revised the Introduction (lines 120–123) to explicitly describe this rationale as part of our methodological motivation, and we have also reiterated this point in the Discussion (lines 571–573) to contextualize the interpretation of decoding results across auditory regions.

We agree that a perfect reconstruction from raw neural signals (*e.g.,* auditory nerve) could, in principle, support the recovery of all feature levels. However, this does not necessarily imply that such features are explicitly represented at each level of the auditory hierarchy. For example, while the auditory nerve may contain sufficient information to reconstruct a sound spectrogram, it does not explicitly encode categorical information such as "speech" or "music" in a form accessible to a linear decoder. Our linear decoding-based framework is therefore intended to probe whether such representations are made explicit and linearly accessible in downstream auditory regions.

To address the concern about statistical significance, we have added CIs for the identification accuracies across ROIs and explicitly stated that the differences observed—such as between A1 and A5—were not statistically significant. These results are now reported in the main text (lines 432–444) and support a distributed, rather than strictly hierarchical, view of auditory processing. Although A1 tended to yield slightly higher decoding fidelity at lower hierarchical stages, the lack of consistent statistical significance tempers the interpretation of strong regional specialization.

(15)

*Lines 437-440 talk about having DNN features (Conv5 vs. FC3) with temporal dimensions leading to "low-level" fidelity of the reconstruction, however this is confounded by a few things. (1) The FC layer is also averaging across *frequency*, and given that the acoustic measures are all time-averaged, it is possible that the information being lost is actually *frequency* specific and not time-specific. (2) I expect that the receptive field for Conv5 is nearly the entire signal due to the hierarchical nature of processing. So even though there is explicitly averaging happening, the time window for Conv5 is already quite large. (3) Conv5 simply has more features than FC3, which might mean that there is more information. (4) From looking at the Figure I'm not sure that the difference between Conv5 and FC3 is statistically significant. Due to all of these, this interpretation should be removed, and the authors should consider controls.
Also, why is only FC3 evaluated? A more natural comparison seems like it should be the first FC layer after Conv5 to make this claim.*

**Response:** We agree that the original interpretation attributing the differences between Conv5 and FC3 to temporal resolution was potentially confounded by several factors, including frequency averaging, receptive field size, feature dimensionality, and the lack of consistent statistical significance. Accordingly, we have retracted the interpretation on lines 437–440 and removed the related discussion from the manuscript.

In line with the reviewer’s suggestion, we additionally evaluated FC1—the first fully connected layer after Conv5—as a more appropriate point of comparison. The results for FC1 showed similar trends to those of FC3 (see R4 Fig), with modest differences relative to Conv5. This analysis is now included in the revised main text (lines 459–460) and presented in S12 Fig.

**R4 Fig. Evaluation of reconstructed sounds from different DNN layers.** Each panel shows the mean identification accuracy calculated for each subject. The dark blue bars represent reconstruction results from Conv5, the orange bars correspond to FC1, and the brown bars represent FC3. Each bar indicates the mean identification accuracy across 50 data points, with error bars showing the 95% CI.

(16)

*The claims of the attention experiment are exaggerated based on the results. The authors claim that two (and in another case three) participants pass a significance threshold, but I am unsure that this threshold is corrected for multiple comparisons.  Additionally, if <1/2 of the participants show this trend, this is not particularly replicable across participants (which is emphasized in [56]). These claims should be toned down/removed.*

**Response:** We agree that the original claims regarding the attention experiment were overstated and did not sufficiently reflect the limitations of the results. In the original analysis, the significance threshold was determined using a binomial test applied independently for each subject, treating subjects as replication units. However, we acknowledge that statistical significance was achieved in only a subset of participants, and the observed effects were not consistent across the subjects.

In response to the reviewer’s comment, we have revised the manuscript to clearly indicate the exploratory nature of this analysis and to temper the strength of the conclusions (lines 50–52). We now explicitly state that only a limited number of subjects reached statistical significance and that these findings require validation in future studies with larger samples (lines 627–628 and lines 642–643). We also removed sentences implying strong or consistent attentional modulation across subjects and reframed the analysis as preliminary evidence of possible attentional effects in some individuals.

(17)

*The author's method relies on a strong generative model to produce the "decoded" stimuli. However, this type of generative model acts as a very strong regularizer for the signal. This regularization may not be appropriate or might be too strong, and this possibility should, at minimum, be discussed.*

**Response:** We agree that the generative model used in our framework functions as a strong regularizer, which may constrain the space of reconstructed signals. Such regularization, while beneficial for producing naturalistic outputs, may also introduce biases that limit the fidelity of reconstructions or cause them to reflect properties of the model rather than true neural representations.

To examine the extent of this effect, we conducted a leave-category-out analysis, where one category (e.g., Music) was entirely excluded from decoder training, then test stimuli of that category were reconstructed. Although this resulted in a modest drop in performance, the model still produced intelligible reconstructions for unseen categories, suggesting that the regularization did not fully restrict generalization. Furthermore, we confirmed that the generator can reconstruct artificially generated sounds not included in the training set, indicating that it is not merely replicating memorized exemplars.

Nevertheless, we acknowledge that the transformer-based audio generator differs fundamentally from biological auditory mechanisms, particularly in its modeling of long-range temporal dependencies. We have revised the manuscript (lines 615–624) to explicitly discuss these limitations and to highlight the need for future work exploring generative architectures that better align with neural processing constraints.

(18)

*Minor:*

*Many of the locations where multiple references are made should be broken up to refer to the specific part of the sentence that the reference applies to, as currently, some of the references seem incorrect. One example is line 53 ([1-5]) where reference [2] does not apply to decoding or imagery, and even though reference [4] applies to imagery, it does not apply to decoding.*

**Response:** We agree that reference groupings should be carefully aligned with the specific claims they support to avoid any potential ambiguity. In response, we have reviewed the manuscript to ensure that all grouped citations are appropriately attributed and clearly mapped to the corresponding parts of each sentence (*e.g.,* lines 60-62).

(19)

*I do not understand line 69-70. Perhaps the authors could rephrase or expand on what they mean by "restricted its use to classification tasks".*

**Response:** We agree that the original phrasing (“restricted its use to classification tasks”) was ambiguous and could lead to confusion. Our intention was to convey that due to the limited temporal resolution of fMRI, previous studies have mainly focused on classification of stimulus categories or decoding of specific acoustic attributes, rather than full-scale reconstruction of continuous auditory stimuli.

To clarify this point, we have revised the sentence on lines 68–71 to more explicitly describe how the temporal constraints of fMRI have shaped the types of decoding approaches commonly used in prior auditory studies.

(20)

*The introduction or discussion could use an explicit explanation of the differences between encoding models and decoding models, and how the differences relate to previous work/this current paper.*

**Response:** We agree that an explicit explanation of the differences between encoding and decoding models is essential for properly contextualizing our approach and clarifying how it relates to prior work.

To address this, we have revised the manuscript to clearly define encoding and decoding models and highlight their complementary roles in auditory neuroscience. Specifically, we note that encoding models aim to predict brain activity from known stimulus features, thereby revealing how the brain represents specific aspects of sensory input. In contrast, decoding models attempt to reconstruct or infer stimuli from measured brain activity, providing insight into the information that is explicitly represented and accessible in neural signals.

We have added this clarification to the revised manuscript (lines 97–111), situating our study within the decoding framework and positioning it as a complementary approach to prior DNN-based encoding studies in the auditory domain.

(21)

*A description of the "codes" should be included in the main text.  For instance, are these continuous? Discrete? Currently, there is a lot of jargon in this section.*

**Response:** We agree that the term “codes” was previously introduced without sufficient explanation, and that the associated section contained too much technical jargon for general clarity.

To address this, we have revised the main text (lines 235–243) to clearly define what we mean by “codes” in the context of our reconstruction pipeline. In our framework, “codes” refer to discrete indices obtained through vector quantization of spectrogram features using the SpecVQGAN model. While the original spectrogram is represented by continuous values, it is encoded into a compact sequence of discrete codebook entries. These discrete codes form a lower-dimensional representation that preserves key spectrotemporal information. The decoder then reconstructs the spectrogram by mapping these codes back into continuous representations, which are ultimately converted into audio waveforms.

We have minimized technical terminology in this revised explanation and provided a visual illustration of the process in S1 Fig to further support reader comprehension.

(22)

*Line 309-310: I'm not sure what "displayed temporally smoothed patterns resembling the original spectrogram" is supposed to convey. Were the original spectrograms smooth as well?*

**Response:** Our intention was to convey that the reconstructed spectrograms appeared more temporally smoothed than the original stimuli—not that the original spectrograms were inherently smooth. This smoothing effect likely reflects limitations in the reconstruction process, which tends to average over fine temporal details.

To clarify this point, we have revised the sentence on line 332 to better demonstrate the properties of the reconstructed spectrograms. The updated text now explicitly states that the reconstructed spectrograms exhibited temporally smoothed patterns poorly relative to the original stimuli.

(23)

*Error bars are missing on SI Fig 7, left plots*

**Response:** The left plots in S7 Fig display the mean profile correlation across units in each DNN layer. Although error bars were included, they were extremely small due to the large number of units and low variance, making them difficult to distinguish in the original figure.

In response to the reviewer’s comment, we have adjusted the plot design to enhance visibility. Specifically, we have enlarged the error bars and refined the visual formatting to ensure they are clearly discernible. These changes have been incorporated into the revised version of the figure.

(24)

*How is the regularization coefficient for the decoder chosen?*

**Response:** The regularization coefficient for the decoder was fixed at 100 and applied uniformly across all ROIs, auditory features, DNN layers, and subjects. This value was determined through preliminary experiments in which we evaluated a range of candidate values to balance decoding accuracy and overfitting. A value of 100 provided stable and consistent performance across conditions. We have added this information to the revised manuscript on lines 855–856.

(25)

*Was the "voxel selection" performed with just the training stimuli? This should be made clear (it is not obvious to me from lines 781-782).*

**Response:** We agree with the reviewer’s comment and confirm that voxel selection was conducted solely within the training dataset to avoid any information leakage into the test set. Specifically, voxel selection was based on the correlation between voxel responses and target features computed within the training dataset only. We have revised the manuscript to clarify this point explicitly (lines 849–850).

(26)

*Why are all voxels not used? This procedure will lead to different voxels being used for different decoding experiments, which seems like an unnecessary confound.*

**Response:** We agree that using different voxel subsets across decoding experiments introduces variability; however, voxel selection is a necessary step to ensure reliable and interpretable decoding results.

First, fMRI datasets typically include a high-dimensional voxel space with relatively few training samples. Including all voxels would significantly increase the risk of overfitting and reduce decoding generalizability. Voxel selection mitigates this by focusing on the most informative voxels for each decoding target, thereby improving model stability and performance.

Second, prior studies have demonstrated that voxel selection not only enhances decoding accuracy but also promotes anatomical interpretability by selecting voxels most functionally aligned with the decoded features (*e.g.,* Yamashita et al., 2008). In our framework, voxel selection was performed independently for each decoding model using the training data only, ensuring no information leakage into the test set.

While this procedure results in different voxel sets across decoding experiments, it is a widely adopted and empirically validated practice in fMRI decoding research. We believe this approach maximizes both decoding accuracy and biological interpretability.

(27)

*The phrase "audio spectrogram" (page 35, 772-774) seems confusing to contrast with a normal "spectrogram". I believe all spectrograms in the paper are technically audio spectrograms.*

**Response:** We agree with the reviewer that all spectrograms used in our study are, by definition, audio spectrograms. The phrase “audio spectrogram” was initially used to distinguish the version used for extracting modulation features, which was computed using different parameters in accordance with prior studies.

However, we acknowledge that this terminology introduced unnecessary confusion. To improve clarity and consistency, we have revised the manuscript to use the unified term “spectrogram” throughout. Where relevant, we now explicitly state when different parameter settings were used (*e.g.,* for modulation feature extraction), without relying on inconsistent terminology. These revisions have been incorporated on lines 843–845.

(28)

*Including references for work highlighting problems with DNN models of the brain could be beneficial for readers. Some examples (but there are numerous lines of work to discuss here):*

[*https://www.sciencedirect.com/science/article/pii/S0893608023001016?via%3Dihub#fig2*](https://www.sciencedirect.com/science/article/pii/S0893608023001016?via%3Dihub#fig2)

[*https://www.nature.com/articles/s41593-023-01442-0*](https://www.nature.com/articles/s41593-023-01442-0)

[*https://arxiv.org/pdf/1801.01944*](https://arxiv.org/pdf/1801.01944)

[*https://arxiv.org/abs/1811.12231*](https://arxiv.org/abs/1811.12231)

[*https://www.biorxiv.org/content/10.1101/2022.03.28.485868v2.full.pdf*](https://www.biorxiv.org/content/10.1101/2022.03.28.485868v2.full.pdf)

**Response:** We agree that addressing the limitations and known challenges of using DNNs as models of brain function is essential for providing a balanced and critical perspective. In response, we have incorporated several of the suggested works into the Discussion section and expanded the relevant paragraph to highlight key concerns—such as sensitivity to adversarial inputs, lack of human-like invariances, and biases stemming from training data constraints.

These additions (lines 591–601) underscore the ongoing gap between artificial neural networks and biological systems, and emphasize the need for future models to better align with the principles of neural computation.

**Reviewer #2**

(1)

*Park et al report an analysis of fMRI data of 5 participants, collected during the listening of 1200 natural sounds that were repeated 4 times (training set) as well as 50 natural sounds repeated 8 times (test set) and an unclear number of superimposed sounds where one of the sounds was to be attended (attention condition).*

*The authors devise a pipeline to decode sound from the neuroimaging data. This pipeline uses multiple deep network modules: (1) a DNN that classifies sounds into one of 309 categories (2) an autoencoder that provides a quantised codebook representation of sounds (3) a chain of models that transforms features from 1 to the codebook from 2, and the spectrograms from the decoder of 2 back to the original waveform. This allows the authors to linearly predict features from a suitable layer of 1 from the fMRI recordings, to then reconstruct the waveform of the sound.*

*The authors apply this pipeline, and then evaluate several comparisons between the reconstructions and the original stimuli in different feature spaces: Spectrograms, features of layers of another pre-trained network called "melception" as well as classic acoustical features such as F0, spectral centroid and harmonic-to-noise ratio. They measure the accuracy with which one would correctly identify the presented stimulus from the decoded stimulus when choosing the stimulus with higher correlation between what is decoded and the original stimuli in the respective feature space.*

*The authors then replace several aspects of their model with alternatives to study the effects: linearly decoding spectrograms or modulation features from fMRI rather than the DNN features, training the DNN only on subsets of sounds, using different auditory regions to decode, using different layers of the DNN to decode and using more compact versions of the DNN pipeline they suggest. Finally, they study how their pipeline works in the attention condition.*

*Altogether, the authors show that their proposed pipeline seems to be the ideal choice compared to the alternatives, that attended aspects of superpositions tend to yield higher correlations with reconstructions than the unattended, and that the model could generalise beyond the distribution of the training set of the DNN (when the training set was limited).*

*Overall, the paper is on a high level of technical sophistication, and comes with many supporting analyses that provide many additional insights into the analyses the authors performed.*

**Response:** We thank the reviewer for their careful review and valuable feedback. Detailed responses to the comments are provided below.

(2)

*Major:*

*First and foremost, the structure could be optimised, where several sections of the results could be entirely relayed to the suppplemental material (certainly those that exclusively refer to supplemental figures) to tighten manuscript.*

**Response:** We agree that the manuscript structure could be improved by streamlining the presentation of results and focusing the main text on the central findings.

In response, we have reorganized the manuscript to more clearly separate primary results from supporting technical details. Specifically, sections that referred exclusively to supplemental figures or presented secondary analyses have been moved to the Supplementary Material.

This restructuring has allowed us to tighten the manuscript, reduce redundancy, and improve readability. The main text now emphasizes the principal findings and their implications, while still guiding interested readers to the corresponding supplementary sections for additional context. We believe these revisions improve the clarity and focus of the manuscript without sacrificing completeness.

(3)

*Secondly, the authors need to work on the neuroscientific claims they intend to make. These seem sometimes unclear and ambiguous (e.g. what we learn about the auditory hierarchy), where I think the authors should spend some more thoughts on how to make more explicit what their results are supposed to show, and how to best communicate that (then start with that question in the corresponding results section, and provide the answer). On other occasions, the authors seem to however also make claims that they cannot inform the reader about, e.g. in the abstract and at the end of the discussion, where the authors talk about insights about "[subjective] perceptual experiences". As such, this manuscript seems mainly like an engineering feat whose neuroscientific contribution is mostly unclear. I am however sure that the authors could rewrite the paper to make it more interesting from a neuroscientific / biological perspective.*

**Response:** We agree that the neuroscientific contributions of the manuscript needed to be stated more explicitly and interpreted with greater care.

In the revised manuscript, we have clarified the scope and basis of our neuroscientific claims in two key areas:

1. Perceptual relevance of decoded representations:
   We acknowledge that the original phrase “subjective perceptual experiences” may have implied claims beyond what the data could support. In the revised version, we now explicitly ground such statements in our behavioral rating experiments. Human raters evaluated the reconstructed sounds in a pairwise identification task, and results showed that reconstructions retained perceptually recognizable features across categories. This supports the interpretation that the decoded DNN features reflect perceptually meaningful aspects of the stimuli, though we refrain from making direct claims about subjective internal experiences.
2. Neural representation and hierarchical organization in auditory cortex:
   While prior encoding studies have demonstrated hierarchical correspondences between auditory cortical regions and DNN layers, our decoding analyses offer a complementary perspective. We employed a linear decoding framework that enables us to assess the explicit availability of information—that is, the extent to which high-level auditory features can be directly recovered from localized brain activity. This approach provides a principled and conservative method to infer what is representationally accessible in each region, consistent with established decoding paradigms in sensory neuroscience. These clarifications and interpretations have been explicitly incorporated into the revised Introduction (lines 120–123) and Discussion sections (lines 570–575) to better align our analytical framework with its neuroscientific implications.

(4)

*Abstract:*

*"Reconstruction from brain activity provides insights into the association between population neural activity and perceptual experiences"*

*This statement seems unclear and highly controversial for entirely unnecessary reasons.*

- *What is reconstructed? Stimulus information? For the first sentence of an abstract, this needs to be made explicit.*

**Response:** We agree that the original phrasing in the abstract was vague and could lead to unnecessary ambiguity. To address this, we have revised the sentence to more clearly define what is being reconstructed. The updated sentence now explicitly refers to the reconstruction of stimulus-related sensory information from brain activity. We have incorporated this revision into the manuscript on lines 36–37.

(5)

*How is "perceptual experience" quantified when merely "reconstructing stimuli from brain activity"? It reads as if this isn't actually quantified, and therefore it is not possible to say anything about the association between population neural activity and perceptual experiences.*

**Response:** We agree that in the original manuscript, the term “perceptual experience” was not clearly defined or linked to an explicit quantification procedure, which could weaken the interpretation of our findings.

To address this, we have clarified in the revised manuscript that perceptual experience was quantified using a behavioral rating task in which human raters judged the similarity between reconstructed sounds and candidate stimuli (S2 Fig; see “Materials & Methods: Identification by human raters”). This pairwise identification task required raters to choose which of two candidates—one being the true stimulus and the other a randomly selected lure—was more similar to the reconstructed sound.

Identification accuracy was consistently above chance across all subjects (e.g., 89.2%, 83.6%, 90.4%, 86.8%, and 90.4% for Subjects 1–5; Fig 4D), indicating that the reconstructions preserved perceptually recognizable features. These results provide empirical support for associating brain activity with perceptually relevant representations.

We have revised the manuscript on lines 44–47 to explicitly describe this behavioral evaluation and ensure that all references to “perceptual experience” are grounded in quantifiable measures.

(6)

*Introduction:*

*"However, their application has mostly been restricted to the classification of predefined speech elements [18,20-22] and the reconstruction of limited words [17,19]. The inherent temporal resolution limitations of fMRI have also restricted its use to classification tasks [23,24]."*

*This discussion of the state of the art should explicitly mention studies by Defossez et al (2022, "Decoding speech perception from non-invasive brain recordings"), which uses MEG and is not restricted to predefined speech elements or the reconstruction of limited words, as well as Tang et al (2023, "Semantic reconstruction of continuous language from non-invasive brain recordings"), which uses fMRI and goes beyond classification tasks.*

**Response:** We agree that including recent work by Defossez et al. (2022) and Tang et al. (2023) provides a more accurate and up-to-date perspective on the current state of auditory decoding research.

Tang et al. (2023) demonstrated that fMRI can be used to semantically reconstruct continuous language, extending its application beyond traditional classification tasks and showing that fMRI-based decoding is not inherently limited to discrete categories. Defossez et al. (2022) showed that MEG recordings, despite lower spatial resolution, can be used to decode speech perception using a contrastive learning objective. However, their method involves selecting from a predefined set of candidates rather than reconstructing arbitrary speech signals, which still imposes constraints on generalizability and reconstruction fidelity.

We have incorporated these studies into the revised manuscript on lines 73–77 to provide a more nuanced view of recent advances while clarifying the remaining limitations that motivate our current approach.

(7)

*"Kell et al. [25] conducted a comprehensive brain encoding analysis, predicting brain responses from the DNN features"*

*This discussion of encoding models based on DNNs should also mention Giordano et al (2023, "Intermediate acoustic-to-semantic representations link behavioral and neural responses to natural sounds"), which is a more modern example of examining hierarchical auditory features in greater detail.*

**Response:** We agree that Giordano et al. (2023) provides an important and contemporary contribution to the literature on DNN-based encoding models, particularly in the context of hierarchical auditory representations.

Their study extends prior work by demonstrating that intermediate layers of DNNs better predict both neural and behavioral responses to natural sounds, effectively bridging low-level acoustic features and high-level semantic content. This offers a refined view of how hierarchical auditory processing may be implemented in the brain.

To reflect this, we have incorporated Giordano et al. (2023) into the revised manuscript alongside the discussion of Kell et al. (2018), providing a more comprehensive overview of relevant encoding studies. This revision has been added to the Introduction on lines 106–108.

(8)

*"found that DNN features more closely resemble neural processing, exhibiting superior decoding performance compared to spectrotemporal features."*

*I don't see a clear, unambiguous mapping from a "superior decoding performance" to the inference of "more closely resemble neural processing". Are the authors aware of the mechanistic claims they are making here? I recommend de-Wit et al, 2016 "Is neuroimaging measuring information in the brain?". As such, the pure decoding is an engineering feat, but not a neuroscientific finding. If the authors claim to elucidate the "neural processing", they have to make it clearer here how they demonstrate more than a good decoding performance.*

**Response:** We appreciate the reviewer’s comment and agree that superior decoding performance alone does not imply a mechanistic equivalence between DNN features and neural processing. Our original wording may have unintentionally suggested a stronger interpretive claim than we intended.

To avoid potential confusion, we revised the Introduction (lines 131–132) to clarify that our claims concern representational alignment between DNN features and brain activity patterns. Specifically, we state that high-level DNN features, which integrate information over broad receptive fields, allow us to reconstruct sounds from fMRI responses without requiring precise temporal alignment between neural activity and auditory input. We further clarified this point in the Discussion (lines 591–601), emphasizing that decoding success reflects alignment in information content, not direct equivalence in processing.

(9)

*"and the effect of ablating training categories"*

*I do not understand at this point what "ablating training categories" is supposed to mean. Do you mean using different sets of categories to be predicted from the sound data? "Ablation" as an analogy to what is done in animal research seems like an unintuitive analogy to restricting the training set.*

**Response:** We agree that the term “ablation,” while common in other research contexts, may not intuitively convey the intended meaning in this setting.

The experiment in question involved systematically excluding one sound category from the training dataset and evaluating the decoding and reconstruction performance on that held-out category. The goal was to assess the model’s ability to generalize to previously unseen sound types.

To improve clarity and accurately reflect the methodology, we have revised the manuscript to replace the term “ablating training categories” with “leave-category-out analysis.” This change has been incorporated into the revised manuscript on lines 139–141.

(10)

*Results:*

*"Decoding performance is evaluated by assessing the capability of the decoded features to identify perceived sounds within the test set."*

*Does this mean you enter the decoded features into the forward pass of the chosen network from the respective layer onwards? Then maybe say so? It's not just the "capability of the decoded features", but more the "capability of the network using the decoded features"?*

**Response:** We agree that the original description may have given the impression that evaluation involved a forward pass through the network, which was not the case.

To clarify, the identification analysis did not involve feeding the decoded features back into the network. Instead, we evaluated decoding performance by directly comparing the decoded features—obtained from brain activity—to precomputed DNN features extracted from the original test stimuli at the same network layer. The similarity between the decoded and candidate features was quantified using Pearson correlation. This allowed us to assess whether the decoded features preserved sufficient information to distinguish the true stimulus from a set of lure candidates.

We have revised the manuscript on lines 207–216 to reflect this more precise explanation and avoid potential confusion about the evaluation procedure.

(11)

*"The binary identification accuracies"*

*Why are they binary? Are they converted into hit / miss? It should be noted somewhere in the main results, preferably early on when the DNN is described between lines 147 / 158 what the optimisation objective was for the DNN -- i.e.the 309 categories.*

*"This assessment entailed comparing the correlation of decoded features with the actual features of the presented stimulus and those of the other test stimuli. Identification accuracy was determined by the number of correctly identified pairs"*

*Does this mean you are seeing which correlation is higher? Please explicitly say how you obtain the "correctly identified pairs".*

**Response:** We appreciate the opportunity to clarify both the identification analysis procedure and the terminology used.

The term “binary identification accuracy” refers to the scoring of each pairwise identification trial as either correct (1) or incorrect (0), based on a simple hit/miss criterion. Specifically, each decoded feature vector was compared to two candidate feature vectors: one corresponding to the true stimulus and the other a randomly selected false candidate from the test set. If the Pearson correlation between the decoded features and the true stimulus features was higher than that with the false stimulus, the trial was marked as correctly identified. Identification accuracy was then calculated as the proportion of correct identifications across all pairwise trials. We have revised the manuscript on lines 207–216 to state this more explicitly.

(12)

*"Audio generator training. First, an autoencoder is trained to compute a compact representation with a size of (5* $\times$ *21) from the spectrogram with a size of (80* $\times$ *336), using codebook alongside the codebook encoder and decoder. Next, an audio transformer is trained to predict sequences of these codebook indices, conditioned on the DNN features, in an autoregressive manner."*

*This is not intelligible and hard to map on the figure. Text describes a procedure with 2 steps, 4A only shows 1 step. 4A shows an autoencoder layout, but is called "generator". Is this the "autoencoder" that is mentioned after "first"? It is unclear what the bar chart on the left is. What is meant by "using codebook alongside codebook encoder and decoder"? What is the decoder-like structure named "D"? What are "DNN features", since everything here (audio transformer, "D", autoencoder, VGGish-ish -- seems to be implemented as a DNN?*

**Response:** We agree that the original description of the audio generator was difficult to follow and did not clearly align with Fig 4A. This was due to inconsistencies in terminology and insufficient explanation of the model components and their interactions.

To address this, we revised the corresponding section in the manuscript (lines 245–252) to clarify the structure and training procedure of the audio generator and ensure consistency with the figure.

The revised version now clearly defines the three major components of the system: an audio transformer that maps decoded DNN features to spectrogram code indices, a codebook decoder that reconstructs spectrograms from those indices, and a vocoder that generates audio waveforms from spectrograms.

We also clarified that the SpecVQGAN-based codebook encoder and decoder, which was trained in advance to compress and reconstruct spectrograms using discrete indices. We replaced this terminology with “codebook system” throughout the manuscript to avoid confusion.

The bar chart on the left side of Fig 4A represents the DNN features decoded from brain activity, which are used as input to the audio transformer. These features condition the transformer to autoregressively generate a sequence of discrete spectrogram indices. The codebook decoder then maps these indices back to a spectrogram, which is finally converted to audio by the vocoder.

These revisions improve consistency between text and figure and clarify the role of each model component in the reconstruction pipeline.

(13)

*"Together, these findings illustrate the ability to approximate both perceptual and quantitative aspects of fidelity and quality as reflected by acoustic features to some extent."*

*What do the authors mean here with "perceptual"? To me, this would need some human evaluations of the reconstructions.*

**Response:** We agree that references to "perceptual" aspects of reconstruction quality should be clearly grounded in human evaluations. To support this, we conducted a behavioral experiment in which human raters assessed the perceptual similarity between reconstructed sounds and candidate stimuli.

The evaluation employed a pairwise identification task (Fig 4D; see Methods: "Evaluation of Reconstruction Quality"), where raters listened to a reconstructed sound along with two candidate sounds—one being the true stimulus and the other a randomly selected lure. Raters were asked to judge which candidate more closely resembled the reconstruction. Reconstruction accuracy, defined as the proportion of correct identifications, was significantly above chance across all subjects, with scores of 89.2%, 83.6%, 90.4%, 86.8%, and 90.4% for Subjects 1 through 5, respectively.

These results demonstrate that the reconstructed sounds preserved perceptual attributes sufficiently well to be reliably distinguished by human listeners. Therefore, our use of the term "perceptual" in describing reconstruction quality is explicitly supported by behavioral data. We have revised the manuscript accordingly on lines 279–296 to clarify this point and ensure that perceptual claims are empirically grounded.

(14)

*Figure S3: What are the decoded features here -- the DNN (Conv5)?*

**Response:** We agree with the reviewer that the decoded features shown in S3 Fig correspond to the Conv5 layer of the DNN. We appreciate the opportunity to clarify this and have revised the figure legend to explicitly state that the features shown were decoded from Conv5.

(15)

*"Although it demonstrated 70% identification accuracy from AC, its performance was lower compared to that using DNN features (S6 Fig)"*

*Was lower where? In the areas other than AC? It is unclear what figure S6 should be compared to.*

**Response:** We agree with the reviewer that the original sentence lacked clarity regarding the basis of comparison. The intended comparison was between the identification accuracy using codebook encoder latent features (shown in S6 Fig) and that using DNN features (presented in Fig 3D), not across brain areas.

However, upon further review, we recognized that this analysis and the associated figure were not central to the main conclusions of the study. To avoid confusion and maintain focus on the primary findings, we have removed S6 Fig and the related discussion from the manuscript.

(16)

*"Quantitative evaluations showed identification accuracies based on spectrogram pixels at approximately 70%. [...]"*

*It could be helpful to refer the reader to figure S5C (brown) here?*

**Response:** We appreciate the reviewer’s suggestion to improve clarity by directing the reader to the relevant figure. In the revised version, we have updated the supplementary figure (S5C Fig) to remove color coding for consistency with the rest of the figure set and to avoid reliance on color for interpretation.

Additionally, to streamline the main text and focus on the primary findings, we have moved the discussion of results involving spectrogram pixel-based decoding—such as the approximately 70% identification accuracy—to the supplementary material. We have revised the manuscript accordingly on lines 330–333 and now refer the reader to S3 Fig for these supplementary results.

(17)

*This whole section ("model components") seems to exclusively refer to supplemental results -- maybe it should be relayed to the supplemental material altogether then?*

**Response:** We agree that the “Model Components” section was largely focused on supplemental analyses and could disrupt the flow of the main results.

To improve the manuscript’s structure and maintain focus on key findings, we have relocated the detailed content of this section to the supplementary material. The most relevant points have been concisely integrated into the main reconstruction section to preserve continuity and provide necessary context. These revisions have been incorporated on lines 335–337 in the revised manuscript.

(18)

*Section hierarchical auditory regions*

*Should be much more centralised in the results / abstract / introduction / rationale.*

**Response:** We agree that hierarchical auditory processing is an important aspect of understanding neural representations of sound and appreciate the reviewer’s suggestion to better reflect its relevance in the manuscript.

In the revised version, we have emphasized the role of hierarchical auditory regions more clearly throughout the manuscript. In the Introduction, we added the rationale that our study aims to investigate hierarchical auditory processing by leveraging linear decoding of brain activity (lines 120–123). This approach allows us to assess the explicit availability of information in each auditory region through simple linear transformations.

Although the differences across regions were not consistently statistically significant, we believe the observed trends offer meaningful insights into the functional organization of auditory processing. Accordingly, we have made the region-wise reconstruction results more visible in the Results section (lines 432–444). In the Discussion (lines 570–576), we explicitly highlighted that our linear decoding approach provides a principled means of interpreting decoding performance across ROIs. This framing supports a cautious but meaningful interpretation of how distributed and potentially hierarchical auditory representations are structured.

By combining this linear decoding rationale with an ROI-based hierarchical analysis, the revised manuscript provides a more coherent and nuanced interpretation of how different auditory regions contribute to sound reconstruction from brain activity.

(19)

*Discussion*

*"Although no discernable difference in feature decoding performance across individual ROIs was observed, there were differences in evaluating reconstructed sound quality (Fig 6A)."*

*It is unclear what is meant by "sound quality" here. Figure 6A shows identification accuracy. How does this map to "sound quality"?*

**Response:** We agree that the term “sound quality” was ambiguous in this context, as Fig 6A presents identification accuracy based on acoustic features rather than a direct perceptual or subjective evaluation.

To improve clarity, we have revised the manuscript to avoid the term “sound quality” in this section and instead refer more precisely to differences observed in the identification accuracy of reconstructed sounds. The revised sentence now explicitly links the result to objective reconstruction evaluations. These changes have been incorporated into the manuscript on lines 548–550.

(20)

*"In contrast, high-level representations were slightly more effective in non-primary regions than in A1. This finding aligns with previous encoding studies [34], which suggest that neurons in the primary auditory cortex vicinity are more inclined towards local integration and favor generic acoustic representations. Meanwhile, neurons in non-primary regions are more involved in longer-term integration and exhibit a tendency for category selectivity."*

*This should mention the work of Giordano et al mentioned above.*

**Response:** We agree that Giordano et al. (2023) offers valuable context for interpreting regional differences in feature representation and auditory hierarchy.

To reflect this, we have incorporated a citation of Giordano et al. into the relevant discussion on lines 582–584. This work supports our observation that non-primary auditory regions tend to favor more abstract, categorical representations, while primary regions are more aligned with low-level acoustic features. The revised text now more clearly links our decoding-based results with complementary findings from encoding studies.

(21)

*"The potential of our method lies in its ability to externalize subjective auditory experience. By bridging the gap between internal cognitive processes and external manifestations, [...]"*

*See above. It is entirely unclear what the authors refer to here with "subjective auditory experience" or "internal cognitive processes".*

**Response:** We agree that the original phrasing using terms such as “subjective auditory experience” and “internal cognitive processes” was overly broad and insufficiently defined in relation to the data presented.

To address this, we have clarified that “subjective auditory experience” refers specifically to the perceptual content of reconstructed sounds, as evaluated through human behavioral identification tasks. These tasks measured how reliably listeners could match reconstructed sounds to their corresponding original stimuli, thereby providing an empirical basis for assessing perceptual resemblance.

We have revised the manuscript accordingly on lines 660–662 to replace ambiguous language with more precise, evidence-based descriptions.

(22)

*Methods:*

*The experimental design section says*

*"This involved a single session with eight runs, each containing 48 attention trials of two sound categories presented simultaneously for 8 s".*

*48 * 8 amounts to 384 trials.*

*The results section says:*

*"During the experiment, subjects listened to 24 superimposed sound pairs from the test dataset under diotic conditions, receiving identical auditory input in both ears, guided by a visual cue. The data collection was repeated eight times, with functional volumes for identical stimuli averaged, yielding 144 test samples for the attention task"*

*Where does the 24 come from (vs 48 in methods section)? Also, where does the 144 come from? This is confusing.*

**Response:** We apologize for the confusion caused by the description of the experimental design and number of data samples.

To clarify, the attention experiment used 24 unique superimposed sound stimuli, with each stimulus composed of two overlapping sounds. For each stimulus, participants were instructed to attend to one of the two component sounds, resulting in two distinct attention conditions per stimulus. This led to 48 unique attention trials (24 stimuli × 2 attention conditions), as stated in the Methods section.

Each of these 48 trials was presented eight times across the session to improve signal-to-noise ratio (SNR). The fMRI responses corresponding to identical stimulus-attention conditions were then averaged across repetitions.

To augment the number of data samples, each 8-s sound stimulus was divided into three overlapping 4-s windows, resulting in a total of 144 test samples (24 stimuli × 2 attention conditions × 3 samples).

We have revised the relevant sections of the manuscript (lines 684–698, 745–750) to reflect this more clearly and consistently.

(23)

*Minor:*

*47: "In a selective auditory attention task, reconstructions tended to resemble the attended sound than the unattended." -- tended to resemble the attended sound more than the unattended?*

*57: "including attention [11] and illusory [12" -- illusory what?*

*58: "highlighted the capabilities of DNN" -- DNN should be in plural here?*

*78: "fMRI scan" -- scan should be plural*

*78: "albeit the advances" -- should be despite the advances?*

*152: "specifically trained for the recognition of natural sounds task" -- specifically trained on the task of recognising natural sounds?*

*420: "(B) Evaluation of reconstructed sounds from different DNN layers, where each layer is denoted by a unique color (ROI: AC). A unique color symbolizes each layer." -- color / layer mapping is explained twice, once is enough.*

*904: "the binary outputs from the three samples of the same stimulus and attention condition were pooed by majority voting to define a single binary data point," -- were pooled?*

**Response:** We have thoroughly reviewed all minor language and formatting issues the reviewer noted, and have implemented the following changes to improve clarity and accuracy:

- Grammatical corrections were made, including revisions such as “resemble the attended sound more than the unattended,” and “specifically trained on the task of recognizing natural sounds.”
- Terminology and formatting were corrected for consistency, including the use of plurals (e.g., “DNNs,” “fMRI scans”) and improved phrasing (e.g., replacing “albeit” with “despite”).
- Ambiguous or redundant expressions were revised or removed, such as eliminating unclear references to “illusory [12]” and redundant figure legend descriptions.
- Typographical errors (e.g., “pooed” corrected to “pooled”) were fixed.
- Phrasing that may have caused confusion, such as references to “sound quality,” was refined for precision.

In a few cases, such as the phrase in line 57, the problematic sentence was removed during revision and no longer appears in the manuscript.

**Reviewer #3**

(1)

*In their manuscript "Sound reconstruction from human brain activity via a generative model with hierarchical auditory features", the authors reconstructed natural sounds from fMRI signals using deep generative models. The authors did an impressive amount of work investigating various aspects of the reconstructions, importance of including different categories of sound in the training, effects of using features from different layers of the neural network and different brain regions to decode from and even the cocktail party conditions. The manuscript would be of valuable contribution to the field of sound reconstruction from brain signals, computational auditory neuroscience and functional neuroimaging overall. Below are a few comments I would recommend the authors address prior to accepting the manuscript for publication.*

**Response:** We thank the reviewer for their careful review and valuable feedback. Detailed responses to the comments are provided below.

(2)

*Major:*

*The authors cite intracranial research (ref. 13 to 19) but only refer to non-invasive methods in the text: EEG and MEG. I recommend clarifying this and explicitly mentioning intracranial work. Especially since there is quite a lot of deep learning used for sound reconstruction there. It may be useful to mention the following recent work:*

[*https://doi.org/10.1371/journal.pbio.3002176*](https://doi.org/10.1371/journal.pbio.3002176)

[*https://doi.org/10.1038/s41586-023-06443-4*](https://doi.org/10.1038/s41586-023-06443-4)

[*https://doi.org/10.1088/1741-2552/ace8be*](https://doi.org/10.1088/1741-2552/ace8be)

*and others.*

**Response:** Thank you for the reviewer’s insightful comment and for recommending these relevant studies. We agree that invasive intracranial research has played a crucial role in advancing speech and sound reconstruction using deep learning approaches, and it should be explicitly acknowledged alongside non-invasive methods such as EEG and MEG.

In the revised manuscript, we have clarified the distinction between invasive and non-invasive approaches and explicitly introduced intracranial work as a key foundation for decoding and reconstruction studies. In particular, we now describe how electrocorticography (ECoG) has been used to reconstruct overt and imagined speech, decode musical content, and apply neural network-based generative models to reconstruct continuous speech from motor and auditory areas.

We have also incorporated references to the recent work the reviewer suggested (e.g., Berezutskaya et al., 2023; Tang et al., 2024; and Angrick et al., 2023) to better reflect the current landscape of intracranial sound reconstruction research. These revisions have been incorporated into the manuscript on lines 77–82.

(3)

*The authors should explain why the correlation of 0.4 is used as reference threshold for their results: "mean profile correlation exceeded 0.4." (lines 175-177).*

**Response:** We agree that the reference to a correlation value of 0.4 could be misinterpreted as implying a predefined threshold of significance or effect size.

To clarify, this value was not used as a formal benchmark but was reported to illustrate the general magnitude of decoding performance across subjects. Our intention was to highlight that profile correlations were consistently positive and of moderate strength, indicating that meaningful information could be decoded from auditory cortical activity.

To avoid confusion, we have revised the manuscript on lines 196–197 to focus on the interpretation of the correlation patterns across conditions, rather than implying that 0.4 served as a specific reference threshold.

(4)

*It would help strengthen the results if the authors provided an objective measure of fidelity for their sound synthesis approach. Currently, they only show some examples of target spectrograms and spectrograms of recovered features (lines 251-265). Would it be possible to quantify the match between these two on a large scale, for example, using the acoustic features described in the paper?*

**Response:** We agree that including an objective measure of fidelity for the sound synthesis component strengthens the evaluation of our reconstruction pipeline.

To address this, we conducted a quantitative analysis of reconstruction fidelity using true DNN features as input to the audio generator, across multiple network layers. This evaluation complements the spectrogram comparisons shown earlier and allows us to isolate the fidelity of the generator itself, independent of decoding accuracy. The results, presented in S13 Fig, show that reconstructions from lower DNN layers (e.g., Conv1–Conv3) closely match the original spectrograms, achieving over 95% accuracy in pixel-based metrics. Reconstructions from Conv4 and Conv5 also maintained high fidelity, with performance around 90%. Notably, higher hierarchical stages 4 resulted in near-perfect reconstruction, suggesting that these features contain sufficient information for accurate sound reconstruction. We have incorporated this analysis into the manuscript on lines 468–473.

(5)

*Could the authors clarify in the Results if the false candidate in the identification analyses was selected at random from the test set? Looking at the example spectrograms, it appears that the acoustic content varies considerably across categories. In identification analyses, were the reconstructed features / sounds mostly compared with false candidates within or outside of their category?*

**Response:** We agree that the selection of false candidates in the identification analysis should be more clearly described.

In our main analysis, each reconstructed feature or sound was compared against all 49 remaining test stimuli, excluding the true stimulus. This means that false candidates were drawn from across the full test set, encompassing both within-category and out-of-category examples. This approach enabled a broad evaluation of identification performance under diverse acoustic conditions.

To address the reviewer’s concern about potential category-driven effects, we additionally conducted a within-category identification analysis, where false candidates were restricted to the same category as the true stimulus. As shown in S3 Fig, reconstructed sounds from the Animals and Environment categories achieved high identification accuracy even within their respective categories. In contrast, Speech and Music reconstructions showed lower within-category identification performance, likely due to greater acoustic similarity and complexity within these categories.

These findings have been incorporated into the revised manuscript on lines 339–344 to clarify the evaluation procedure and contextualize the results.

(6)

*The authors refer to some of the acoustic features they use for assessing reconstructions as representing "perceptual […] aspects of fidelity" (line 290). I recommend that the authors clarify which features they refer to. The paper does not contain results using human judgments of perceptual quality of the reconstructions. To what extent can they ensure that the acoustic features they focus on represent perceptual quality of sounds?*

**Response:** We agree that referring to certain acoustic features as representing "perceptual aspects of fidelity" requires clarification and empirical support.

To empirically validate the perceptual relevance of our reconstruction results, we conducted a behavioral rating experiment (S2 Fig; see "Materials & Methods: Evaluation of Reconstruction Quality"). In this pairwise identification task, human raters were asked to choose which of two candidate stimuli more closely matched each reconstructed sound. Identification accuracy was significantly above chance across all subjects (e.g., 89.2%, 83.6%, 90.4%, 86.8%, 90.4% for Subjects 1–5; Fig 4D), and category-specific results showed strong performance for Animals and Music, and robust identification for Speech and Environment as well.

These behavioral findings support the interpretation that the reconstructed sounds retained perceptually meaningful features, and they provide empirical grounding for our use of the term “perceptual aspects of fidelity” in relation to both human judgments and selected acoustic features. We have clarified this point in the revised manuscript on lines 279–296.

(7)

*To what extent do the authors compare apples to apples when comparing sound reconstruction quality across DNN features, spectrograms and modulations given that different methods are used to synthesize sound from these features (lines 304-315)?*

**Response:** We agree that earlier comparisons across feature types may not have ensured a fully controlled evaluation, as each feature was originally reconstructed using a different synthesis approach.

To address this, we conducted a controlled analysis in which we kept the entire reconstruction pipeline—including the audio generator architecture—constant and varied only the input feature type. Specifically, we replaced the DNN features with either spectrogram or modulation features, while using the same decoding model and generator structure across conditions. This allowed us to directly assess reconstruction performance attributable to each feature type under matched conditions (see S4 Fig and S3 Movie).

The results showed that spectrogram- and modulation-based reconstructions preserved some coarse spectrotemporal patterns but lacked the finer detail present in DNN-based reconstructions. Spectrogram-based reconstructions achieved approximately 70% identification accuracy, and while modulation features slightly outperformed spectrograms, they remained inferior to DNN features. Notably, modulation-based reconstructions degraded more substantially at higher hierarchical stages, highlighting the role of deep, task-optimized representations in preserving complex auditory information.

These results have been incorporated into the revised manuscript on lines 333–335 and further detailed in S4 Fig and S3 Movie.

(8)

*I am not sure how convincing is the argument the authors make about robustness of reconstructions for speech and music in the ablation study (lines 333-337). Earlier the authors mention the perceptual discrepancy in the reconstruction at least for music (lines 326-327). It appears more likely that the quantitative metrics the authors are using (possibly except for high melceptron) do not capture the perceptual quality of the reconstructions.*

**Response:** We agree that our original description of the robustness of reconstructions for speech and music in the leave-category-out analysis may have overstated the conclusions, especially in light of perceptual discrepancies noted elsewhere in the manuscript.

While quantitative metrics indicated moderate retention of performance after excluding categories such as music and speech, visual inspection revealed clear perceptual degradation, particularly for music. Reconstructed spectrograms retained some rhythmic or melodic structure, but also showed increased noise and reduced clarity. These findings suggest that the quantitative metrics used—while useful for coarse comparisons—may not fully capture perceptual fidelity, especially for complex, temporally structured sounds. Among the evaluated features, only higher-layer Melception features partially aligned with perceptual impressions, though even they did not fully account for the observed degradation.

To better reflect these limitations, we have revised the manuscript to temper the original claims and explicitly acknowledge the mismatch between quantitative evaluation and perceptual experience. The updated description more accurately characterizes the limits of our approach and the need for more perceptually aligned metrics or feature representations. These revisions have been incorporated into the manuscript on lines 403–406 and 413–415.

(9)

*Lines 410-412: "Conversely, assessments with intermediate and high-level hierarchical features revealed that reconstructions from the auditory …": it is not clear to me what the authors refer to as "intermediate and high-level hierarchical features". It is not evident from Fig 6A that in any of the features A4 scores higher than early processing regions.*

**Response:** We agree that the original phrasing was unclear and could be interpreted as suggesting that non-primary auditory regions such as A4 consistently outperformed early regions like A1, which is not supported by the data in Fig 7A.

Our intent was to highlight that while primary regions like A1 tend to perform better for low-level features, some non-primary areas (e.g., PBelt) exhibited comparable performance at intermediate and high hierarchical stages. However, this pattern was not consistent across all non-primary regions, and A4 in particular did not reliably exceed the performance of early regions.

To avoid misinterpretation, we have revised the manuscript to describe these trends more accurately and conservatively. The updated text on lines 434–435 clarifies that differences across ROIs were generally small, and no consistent advantage was observed for higher-level regions across all feature types.

(10)

*Minor:*

*Typo on line 293: should be "we" instead of "w"*

*Please increase the resolution of Figure 5*

*The manuscript would benefit from additional proof reading.*

**Response:** We have addressed the following points in the revised manuscript:

- Line 293 typo: The typographical error has been corrected by replacing “w” with “we.”
- Figure 5 resolution: The resolution of Figure 5 has been increased to improve clarity and readability.
- Proofreading: The entire manuscript has undergone an additional round of proofreading to correct minor typographical issues and improve overall clarity and presentation.

We appreciate the reviewer’s attention to these details, which helped us refine the manuscript for better readability and presentation quality.
