# Supplementary material for: Natural sounds can be reconstructed from human neuroimaging data using deep neural network representation
Source: PLoS Biol. 2025 Jul 23;23(7):e3003293. doi: 10.1371/journal.pbio.3003293 (PMC12313072; doi:10.1371/journal.pbio.3003293)
Supplement: Attachment — Submitted filename: Response to Reviewers.docx [file pbio.3003293.s032.docx]

Responses to reviewers: “Sound reconstruction from human brain activity via a generative model with hierarchical auditory features”

We sincerely thank the editor and reviewers for their constructive feedback, which has greatly helped us improve the clarity and quality of the manuscript. We carefully considered all comments and made corresponding revisions throughout the paper. Below, we provide detailed point-by-point responses.

(Reviewers’ comments in italics)

**Reviewer #1**

(1)

*I thank the authors for making a substantial effort to improve the quality of the paper. Overall, the thorough revisions and additions have strengthened the contribution. I especially appreciated the effort taken to run a behavioral study on the generated sounds.  For the most part, my concerns have been well addressed, and I found the paper a fascinating read and an impressive engineering feat. 
A few remaining things that I believe would help the paper before publication, which can be addressed with fairly minor wording changes:*

**Response:** We thank the reviewer for their thoughtful and constructive feedback. We are pleased that the revised manuscript was received positively, and we appreciate the recognition of contributions. Below, we respond in detail to each of the remaining comments and describe the corresponding changes made in the manuscript.

(2)

*I still don't think the claim of lack of hierarchical correspondence for decoding (line 566-568) is appropriately backed up by the presented results.  Is this statement just referring to Figure S10A, with the single layer of the DNN decoding each region? Overall, lines 566-576 seem very bold to be making from just this result, and one would have to do substantial control analysis beyond what is currently in the paper to make sure that there are not additional confounds. I encourage the authors to significantly tone down this section.*

**Response:** We agree that our original statement regarding the lack of hierarchical correspondence in decoding may have overstated the implications of our results. Our intention was to highlight that decoding performance and reconstruction quality did not differ substantially across individual auditory ROIs, suggesting that the relevant information is distributed across multiple regions of the auditory cortex. However, we acknowledge that framing this as a lack of hierarchical correspondence could be misleading, particularly in the absence of more extensive control analyses. In response to this concern, we have revised the corresponding section in the Result (lines 443–446) and Discussion (lines 568–574 and lines 584–589) to avoid overgeneralization and to present a more cautious interpretation focused on the distributed and linearly accessible nature of auditory representations revealed by our decoding approach.

(3)

*It is still not clear how the reconstruction of input provides "crucial insights" into neural coding (first line of the introduction, lines 57-58). This was also brought up in Reviewer 2's concern #8. I appreciate the authors' response to my concern #14; however, the response addresses linear decoding, which is not what the reconstruction process is doing. The main text now seems generally clear what the scientific contribution of the decoder vs. the reconstruction says, but I think some of the abstract and introduction should be modified with this in mind (i.e. first sentence mentioned above).*

**Response:** We thank the reviewer for pointing out the ambiguity in the original opening sentence of the Introduction. As suggested, we have revised the sentence (lines 58–60) to clarify the role of reconstruction in the context of neural coding. The updated sentence now describes reconstruction as a tool to investigate how population-level neural activity encodes coherent perceptual experiences. This revision distinguishes the function of reconstruction from that of decoding and ensures that the contribution is framed more appropriately within the scope of representational analysis.

(4)

*The identifiability result with textured and scrambled stimuli at different lengths provides interesting insights into how the reconstructions are performing, however result this is not well captured in the abstract (the line "despite its ability to reconstruct exact temporal sequences" on 52 seems vague). I appreciated the authors interpretation of this result in the discussion and relating it to the nature of the fMRI signal, so perhaps using some similar wording in the abstract would help with clarity.*

**Response:** We thank the reviewer for the thoughtful suggestion to better reflect the interpretation of the temporal fidelity analysis in the abstract. In the revised version (lines 52–55 and lines 605–616), we clarified that the reconstructed sounds preserve short-term acoustic structure but lack longer temporal sequences, consistent with the temporal limitations of the fMRI signal. This revision aligns with the interpretation presented in the Discussion.

(5)

*For the category specific analysis of the behavioral results (lines 292-294), is the randomly selected alternative always from the same category? Or is this just the average across the same and different categories (I think it is probably this)? As written, it is a little unclear in the main text.*

**Response:** As you correctly inferred, the category-specific behavioral results in lines 292–294 were based on identification performance averaged across both within-category and across-category trials. We have revised the text to clarify this point in the main manuscript (lines 293–295).

Additionally, we conducted a separate within-category analysis in which both the true stimulus and the selected lure belonged to the same category. The results of this analysis are presented in Fig S6.

(6)

*Also, regarding the behavioral results, can any within-category confusions be reported (i.e. something similar to Fig S6 for the modeling results). For instance, it seems likely that a speech sound is easy to tell apart from thunder, but then the identification of one speech sound from another speech sound is close to chance. This would be good to know and would help the interpretation of the behavioral results.*

**Response:** We thank the reviewer for this insightful comment. In fact, the left panels of Fig S6 provide a within-category analysis of the behavioral identification results based on human ratings. These results indicate that within-category discrimination is generally feasible: human ratings are consistently above 70% for animal and environment sounds, and between 60–80% for speech. Music sounds exhibited greater variability across participants, suggesting more inconsistent perceptual reconstruction in that category.

These findings imply that some degree of fine-grained differentiation within the speech category is possible for human raters, although less reliable than cross-category distinctions. In contrast, the variability in music category ratings suggests a relative lack of detail in reconstructed features for that category. We have clarified this point in the revised text to aid interpretation of the behavioral results (lines 341–346).

(7)

*I do not think there is enough data to validate lines 326-328. This observation could perhaps be mentioned in the discussion as a future direction, but it seems inappropriate for the results.*

**Response:** We agree with the reviewer that the original statement in lines 326–328 may have overstated the implication of our results. In the revised manuscript (lines 328–329), we clarified that it showed relatively higher correspondence with human judgments compared to other objective evaluation metrics.

(8)

*Error bars are not included in Fig 8C. If there is a reason they are not included (for instance if they are too small as mentioned in the rebuttal for other plots), then this should be mentioned in the caption, especially for the human ratings.*

**Response:** In the attention test (Fig 8C), identification for each trial was scored as a binary outcome based on whether the attended or unattended stimulus was judged to be more similar to the reconstructed sound. Since the results are based on binary outcomes at the sample level and not continuous variables, we used a significance threshold line (based on a binomial test) instead of conventional error bars. We have added this clarification to the figure legend to explain the rationale for not including error bars, particularly for the human rating results (Fig 8. caption).
